# A critical role for *Vibrio parahaemolyticus* LPS to mediate evasion of host immune response during infection

**DOI:** 10.1073/pnas.2426547122

**Published:** 2025-08-13

**Authors:** Jananee Jaishankar, Hyojik Yang, Ian P. O’Keefe, Brandon M. Tenaglia, Lisa N. Kinch, Robert K. Ernst, Kim Orth

**Affiliations:** ^a^Department of Molecular Biology, University of Texas Southwestern Medical Center, Dallas, TX 75390; ^b^HHMI, Dallas, TX 75390-9148; ^c^Department of Microbial Pathogenesis, University of Maryland, Baltimore, MD 21201; ^d^Department of Biochemistry and Molecular Biology, University of Maryland, Baltimore, MD 21201; ^e^Department of Biochemistry, University of Texas Southwestern Medical Center Dallas, TX 75390

**Keywords:** T3SS, *Vibrio*, LPS, Lipid A, TLR4

## Abstract

*Vibrio parahaemolyticus* (*V. para*), a Gram-negative halophilic bacterium, is the leading cause of seafood-borne gastroenteritis. While the cytotoxic and enterotoxic capabilities of this bacterium have been previously investigated, the strategies it employs to circumvent innate immune detection by the host remain poorly understood. Bacterial lipopolysaccharide (LPS) serves as the major pathogen-associated molecular pattern that triggers host inflammatory responses, and its lipid A structure contributes to the degree of innate immune system activation. We sought to define the structure of the lipid A component of LPS in *V. para* and probe its role in host innate immunity. Free living *V. para* synthesizes a predominantly hepta-acylated lipid A structure, while also producing a minor subset of hexa-acylated lipid A. This altered acylated lipid A elicits a weakened TLR4 immune response as compared to *Escherichia coli*, allowing *V. para* entry and replication inside host cells. Deletion of the *V. para* lipid A biosynthesis enzyme *lpxM* (VP0213) leads to a mutant strain with altered lipid A secondary acylation, enhanced immune response, and impaired replication within epithelial cells. This study provides evidence that the unique LPS structure synthesized by *V. para* plays a crucial role in evading host innate immunity, facilitating the bacterium’s survival and replication within host cells.

## Significance

*Vibrio parahaemolyticus* (*V. para*) infections are usually self-limiting but can lead to sepsis in immunocompromised patients. As a key component of the Gram-negative bacterial outer membrane, lipopolysaccharide (LPS) protects bacteria from toxic molecules and allows their survival under stressful conditions. We show that extracellular *V. para* synthesizes a predominantly hepta-acylated lipid A component of LPS, protecting the bacteria from host recognition. Bacteria produce hexa-acylated lipid A during intracellular replication. Altering lipid A acylation through deletion of the acyltransferase *lpxM* affects *V. para* pathogenicity, providing evidence that LPS plays a key role during infection. This study highlights the potential for targeting LPS as a therapeutic strategy, suggesting a unique avenue for developing treatments against infections caused by *V. para*.

*Vibrio parahaemolyticus* (*V. para*) is a Gram-negative bacterium that is the leading cause of seafood-borne gastroenteritis (1). In some cases, infections can also lead to sepsis (2). Many virulence factors, including hemolysins, two type 3 secretion systems (T3SS1 and T3SS2), type 6 secretion systems, and outer membrane proteins, have been implicated in its pathogenesis (3). Our group has previously demonstrated that *V. para* exhibits an intracellular lifestyle-mediated by T3SS2 (4), which plays a key role in intestinal pathology (5). The T3SS2 is activated by bile salts, specifically taurodeoxycholate (TDC) (6–8). Although several effectors of T3SS2 and their roles in intracellular infection have been characterized (9–12), limited information is available on the role of many other virulence factors of *V. para,* such as the lipopolysaccharide (LPS) during infection.

**Fig. 1.**
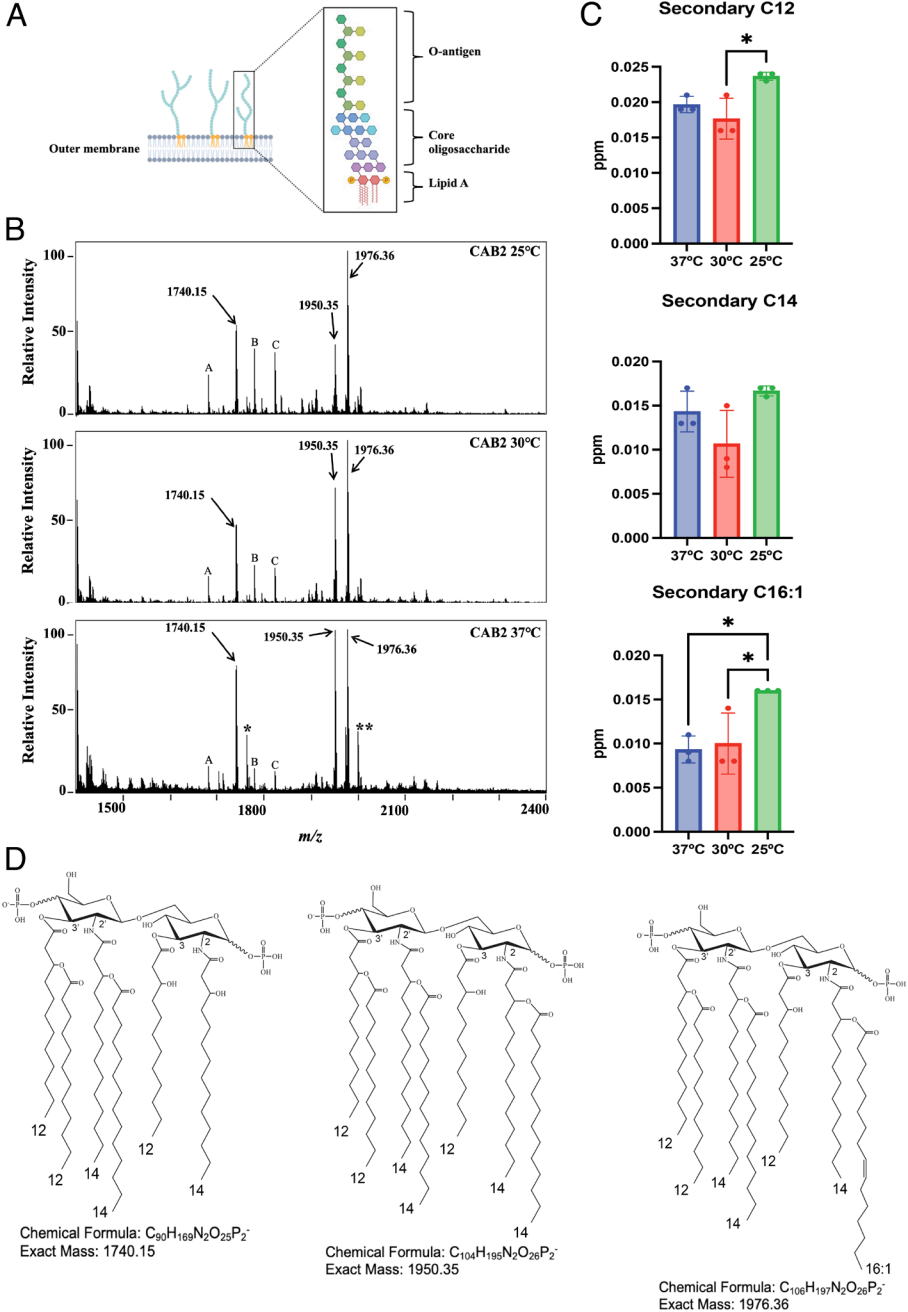
Lipid A structure of *V. para.* (*A*) Schematic showing the typical structure of LPS embedded in the outer membrane of bacteria that composed of the O-antigen subunits, core oligosaccharide, and lipid A (created in BioRender). (*B*) FLAT-MS spectra collected on a Bruker timsTOF (trapped ion-mobility spectrometry time-of-flight mass spectrometry) of *V. para* CAB2 grown in minimal media supplemented with MgSO_4_ at 25, 30, and 37 °C. At all these temperatures, CAB2 produces lipid A peaks at *m/z* 1740.15, 1950.35, and 1976.36. A, B, and C correspond to unidentified peals with *m/z* 1680.56, 1778.53, and 1822.52; * corresponds to sodium (Na) adducted lipid A ion at *m/z* 1740.15 and ** corresponds to sodium (Na) adducted lipid A ion at *m/z* 1976.36. (*C*) Distribution of acyl chains C12, C14, and C16:1 in the CAB2 strain grown at 25, 30, and 37 °C. Statistical significance was calculated using one-way ANOVA. (*D*) Chemical structure of the hexa-acylated (*m/z* 1740.15) and hepta-acylated (*m/z* 1950.35, 1976.36) lipid A of *V. para* CAB2 strain.

LPS is embedded in the outer membrane of Gram-negative bacteria and consists of three components: the O-antigen, core oligosaccharide, and lipid A (Fig. 1*A*). These components are essential to maintain the structural integrity of the bacterial outer membrane (13). The O-antigen consists of repeated oligosaccharide subunits of varying lengths that contribute to the antigenic specificity of the molecule. The core oligosaccharide connects the O-antigen to the lipid A membrane anchor. In *Escherichia coli,* lipid A is composed of a β-(1′,6)-linked disaccharide of D-glucosamine phosphorylated at the 1 and 4′ carbon positions (14). The disaccharide is hexa-acylated and contains four C14 hydroxy acyl chains attached at the 2,3,2′,3′ carbon positions, as well as acyloxyacyl (secondary) acyl chains—one C14 acyl and one C12 acyl, attached to the beta hydroxy groups (15). As the structure of LPS is mostly conserved in Gram-negative bacteria, it acts as an important pathogen-associated molecular pattern (PAMP) recognized by the host innate immune system (16). However, considerable structural variations in LPS among different bacteria affect host–pathogen interactions during an infection.

LPS, commonly referred to as endotoxin, is synthesized at the inner membrane of the bacterial cell. Following its production, it is transported to the outer membrane through a specialized system known as the Lpt system, which facilitates the movement of LPS essential for maintaining the integrity of the bacterial outer membrane (17). The Raetz pathway outlines a sequence of enzymes—specifically LpxA, LpxC, LpxD, LpxH, LpxB, LpxK, WaaA, LpxL, and LpxM that perform unique functions in the biosynthesis of lipid A (18). The final step in lipid A biosynthesis is performed by the late acyltransferases LpxL and LpxM. These enzymes add a C12 acyl chain and a C14 acyl chain, respectively, resulting in the formation of a hexa-acylated lipid A species in *E. coli* (19). However, many bacterial species exhibit variations in the substrate specificity of their late acyltransferases. This variability can influence the acyl chain length, saturation, and branching, as well as the number and positions of the added secondary acyl chains (18). Heterogeneity in the lipid A structure has been shown to differentially modulate the host immune response (20). While many bacterial species, including *E. coli*, *Salmonella typhimurium*, *Vibrio cholerae*, and *Legionella pneumophila* are known to synthesize hexa-acylated lipid A, certain species, such as *Yersinia* spp. exhibit a temperature-dependent acylation pattern. Specifically, these bacteria produce hexa-acylated lipid A at 26 °C but shift to synthesizing tetra-acylated lipid A when grown at the higher temperature of 37 °C, which is the typical temperature for mammalian hosts (14). Similarly, tetra-acylated lipid A is synthesized by *Francisella tularensis* (16) and *Coxiella burnetii* (21). In addition, several bacteria have also been reported to hyperacylate their lipid A, including *Pseudomonas aeruginosa* (22), *Klebsiella pneumoniae* (23), and *Salmonella enterica* (24). Specifically, *S. typhimurium* consists of a C16 fatty-acyl chain linked to the primary acyl chain at the C-2 position, which is facilitated by the enzyme palmitoyl transferase PagP (25).

Mammalian cells employ two primary mechanisms for sensing lipid A. The first involves the recognition of extracellular lipid A by the Toll-like receptor 4/myeloid differentiation factor 2 (TLR4/MD2) complex located on the surface of immune and epithelial cells. Upon lipid A binding to this complex, it triggers dimerization, leading to the activation of NF-κB and subsequent induction of proinflammatory cytokines. The second mechanism occurs when LPS/lipid A is detected inside the cell by Caspase-4/5 and Caspase 11 in humans and mice, respectively through their caspase activation and recruitment domain (CARD). This interaction results in the oligomerization of Caspase-4/11 and the cleavage of Gasdermin-D, producing a fragment that forms pores in the plasma membrane, ultimately leading to pyroptosis and the release of IL-1β (26). The diverse structures of bacterial lipid A can result in differential recognition by either the TLR4/MD2 or Caspase-4/5 complexes in the host, which can trigger activation or evasion mechanisms. This distinction plays a crucial role in determining whether the immune response will facilitate the clearance of the bacteria or contribute to their pathogenicity.

Some research groups have attempted to elucidate the biosynthesis of LPS and determine the lipid A structure in *V. para* (27–32), but discrepancies in the reported length of acyl chains exist between different groups. Moreover, there are no studies devoted to understanding the role of the different *V. para* lipid A structures in inducing the host immune response. In the present study, we have determined the lipid A structure of an extracellular *V. para* CAB2 strain and compared it to the lipid A structure from intracellular bacteria that have invaded epithelial cells. We report that *V. para* synthesizes a mixture of hexa- and hepta-acylated lipid A structures when grown extracellularly and synthesizes hexa-acylated lipid A during replication in epithelial cells. Wild-type *V. para* is seemingly unrecognized by TLR4, whereas a knockout strain encoding a deletion of the secondary acyltransferase *lpxM* (CAB2Δ*lpxM*) is sensed by TLR4. Additionally, CAB2Δ*lpxM* strain shows reduced invasion, with intracellularly replicating bacteria exhibiting a filamented phenotype, indicative of stress, inside epithelial cells (11). Overall, this study indicates that *V. para* uses its unique lipid A as a disguise during the infection process. When this lipid A structure is compromised, the bacteria can no longer hide from innate immune detection and are cleared by the host.

## Results

### *V. para* Synthesizes Hexa- and Hepta-Acylated Lipid A Species.

**Fig. 2.**
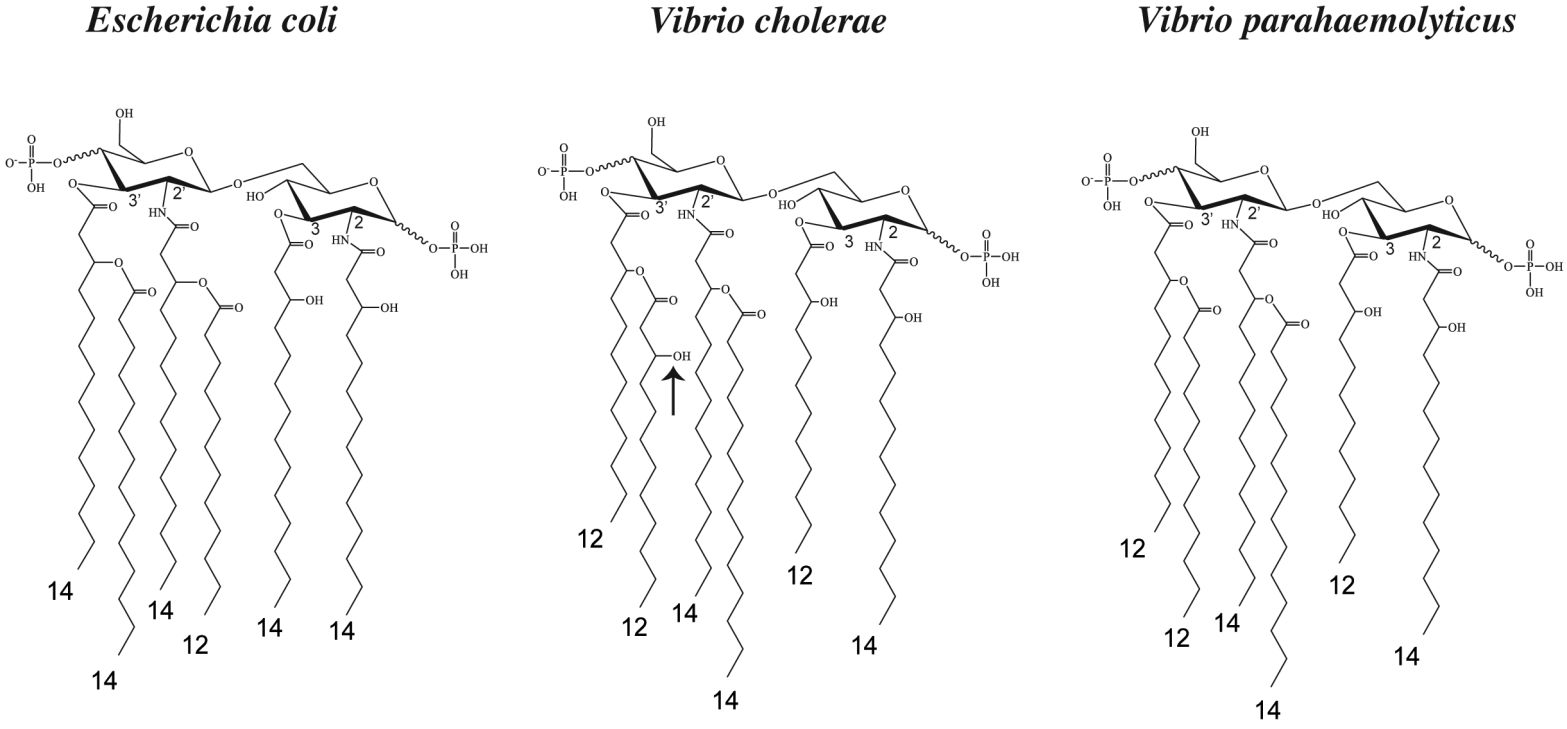
Structure of hexa-acylated lipid A of *V. para* (and *V. cholerae*) is different from *E. coli.* Comparison of the lipid A structures of *V. para* with *E. coli* and *V. cholerae.* indicate that *Vibrio* spp. synthesize equal ratios of C12 and C14 acyl chains in their lipid A contrary to other bacteria, which synthesize a 1:5 ratio of C12:C14 acyl chains. The arrow indicates the presence of hydroxyl group in the secondary acyl chain at the 3′-position in *V. cholerae* lipid A that is absent in *V. para*.

The structure of lipid A in Gram-negative pathogens can vary when bacteria are grown at different temperatures. To determine whether *V. para* generates different lipid A structures when grown at different temperatures, we employed fast lipid analysis technique (FLAT) (33) to analyze the lipid A structure of the invasive *V. para* CAB2 strain (Table 1) grown at 25 °C (ocean temperature), 30 °C (optimal growth temperature), and 37 °C (mammalian host). At all temperatures, three major lipid A peaks were observed at *m/z* 1740.15, 1950.35, and 1976.36 (Fig. 1*B*). The amount of C12, C14, and C16:1 acyl chains in CAB2 lipid A grown at the three different temperatures were also determined by gas chromatography paired with flame-ionization detection (GC-FID), which indicated a greater amount of C12, and C16:1 acyl chains at 25 °C as compared to 30 and 37 °C (Fig. 1*C*). To deduce the chemical structure of each lipid A precursor ion, tandem mass spectrometry FLAT (FLAT^n^) was used (*SI Appendix*, Fig. S1 *A*–*C*). The peak at *m/z* 1740.15 corresponds to hexa-acylated lipid A containing three C12 and three C14 acyl chains. The peaks at *m/z* 1950.35 correspond to hepta-acylated lipid A with three C12 and four C14 acyl chains; and *m/z* 1976.36 corresponds to hepta-acylated lipid A containing three C12, three C14, and one C16:1 acyl chain (Fig. 1*D*). Next, we determined whether individual lipid A structures were strain specific. FLAT analysis of a *V. para* environmental strain (A2), a *V. para* AHPND strain (D4) (34), the clinical isolate RIMD2210633, and its four laboratory-adapted derivatives POR1, CAB2, CAB3, and CAB4 with different pathogenicity determinants (Table 1) was carried out. All tested strains showed the *m/z* peaks at 1740.15 (hexa-acylated), 1950.35, and 1976.36 (hepta-acylated), suggesting conserved lipid A structures among the strains (*SI Appendix*, Fig. S2*A*). The classic lipid A structure of *E. coli* (and *Shigella flexneri* and *S. typhimurium*) contains one C12 and five C14 acyl chains while the lipid A in *Vibrio* species contains three C12 and three C14 acyl chains (Fig. 2). Interestingly, the hexa-acylated lipid A structure of *V. para* is very similar to that of *V. cholerae,* except for a hydroxyl group in the secondary C12 side chain linked to the 3′-position in *V. cholerae* (35).

**Table 1.** Summary of *V. para* strains used in the study

Strain	Hemolysins	T3SS1	T3SS2	Insect toxin	Reference
RIMD2210633	+	+	+	−	(66)
POR1	−	+	+	−	(61)
CAB2	−	−	+	−	(67)
CAB3	−	+	−	−	(67)
CAB4	−	−	−	−	(67)
Non-AHPND strain A2	−	+	−	+	(34)
AHPND strain D4	−	+	−	+	(34)
CAB2Δ*lpxM*	−	−	+	−	This study
CAB2Δ*lpxM*+pBAD-*lpxM*	−	−	+	−	This study

### Intracellular *V. para* Synthesizes a Hexa-Acylated Lipid A Structure.

**Fig. 3.**
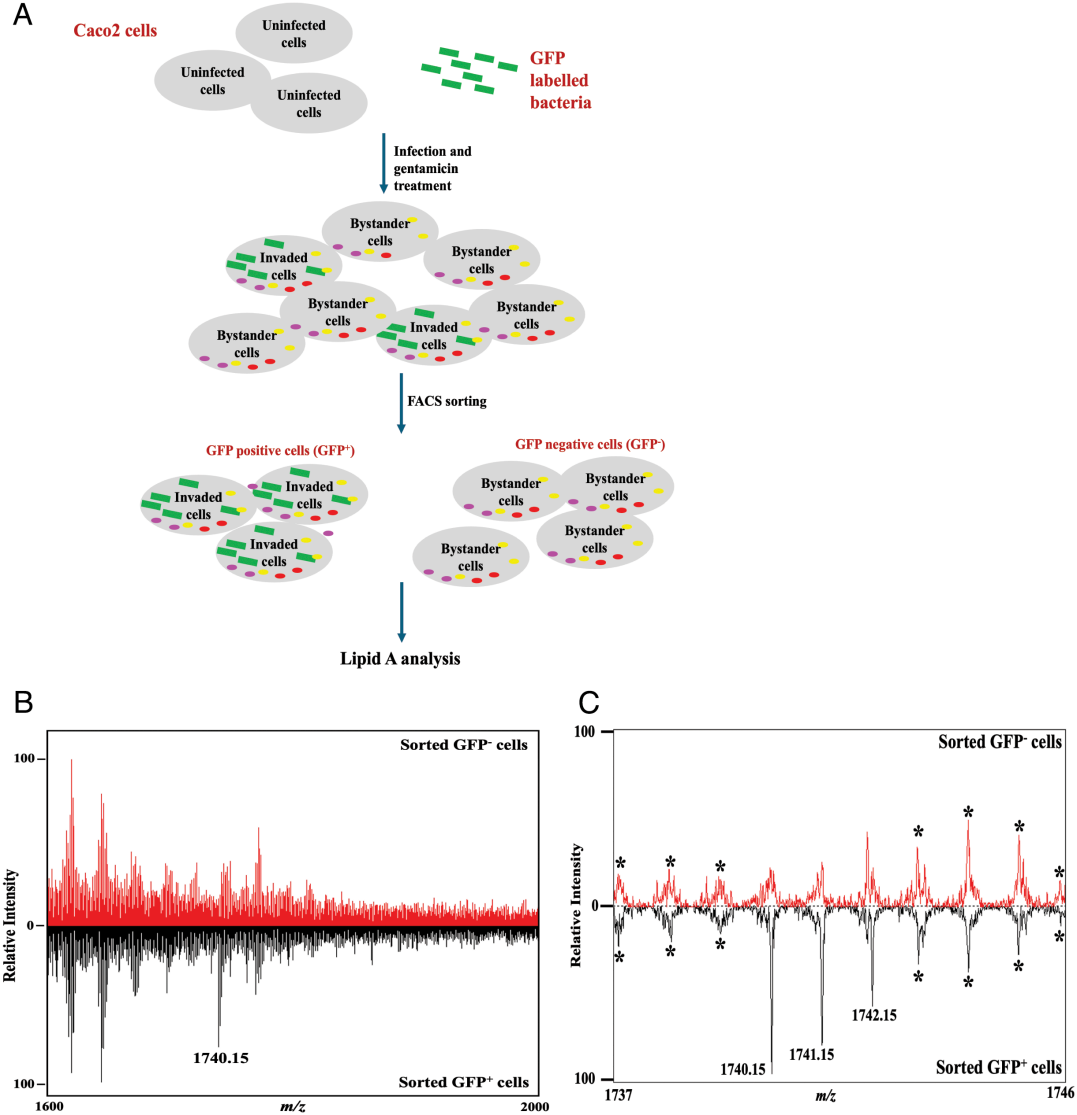
Structure of intracellular *V. para* CAB2 lipid A structure determined by FLAT^n^. (*A*) FACS methodology to collect intracellular bacteria from Caco-2 cells. CAB2 strain of *V. para* expressing GFP was used to infect Caco-2 cells, followed by gentamicin treatment to kill extracellular bacteria. After 4 h of gentamicin treatment, the invaded (GFP^+^) and bystander (GFP^−^) cell populations were collected using a cell sorter to select for GFP^+^ and GFP^−^ cells.; (*B*) FLAT-MS spectra of lipid A in sorted invaded (GFP^+^ cells) and bystander (GFP^−^ cells) Caco-2 cell population collected using a Bruker timsTOF Flex. The peak at *m/z* 1740.15, detected only in the invaded cell population (GFP^+^ cells), corresponds to hexa-acylated lipid A. (*C*) Zoomed in FLAT-MS spectra from *m/z* 1,737 to 1,746 indicate that the peak at *m/z* 1740.15 is exclusive to GFP^+^ cells and is not observed in the GFP^−^ cells. The adjacent peaks observed at *m/z* 1741.15 and 1742.15 correspond to the M+1 and M+2 isotopic variants, respectively. * Correspond to peaks present in both cell populations (GFP^+^ and GFP^−^).

*V. para* has been shown to exhibit an intracellular lifestyle by invading and replicating in the cytosol of nonphagocytic cells (4). To specifically look at bacteria inside host cells, we used the CAB2 strain of *V. para* (Table 1) that expresses and secretes effectors using T3SS2 to invade and replicate inside host cells. Using a constitutive GFP expressing plasmid in CAB2 strain (CAB2-GFP) for infecting Caco-2 cells, we first verified that expression of GFP does not change the lipid A profile of bacteria without host cells (*SI Appendix*, Fig. S2*B*). Next to understand whether the bacteria modify their lipid A structure during replication inside Caco-2 cells, we developed a fluorescence-activated cell sorting (FACS) protocol to separate and collect intracellular bacteria from invaded host cells (Fig. 3*A*). This separation revealed a major lipid A species at 1740.15 *m/z* exclusively in the GFP-positive cells (GFP^+^) (Fig. 3*B*). Accurate mass analysis of the singly charged, deprotonated monoisotopic anions of lipid A was performed. The experimentally determined *m/z* values matched theoretical predictions to two decimal places, enabling ion characterization. Within the *m/z* range of 1,737 to 1,746, the magnified mass spectrum illustrates that the hexa-acylated lipid A (C90H169N2O25P2−, monoisotopic [M] at *m/z* 1740.15) is uniquely identified in GFP-positive cells. Furthermore, the adjacent peaks observed at *m/z* 1741.15 and 1742.15 correspond to the M+1 and M+2 isotopic variants, respectively, which arise from the natural abundance incorporation of single and double 13C atoms (Fig. 3*C*). All other peaks detected in these samples do not correspond to any known lipid A structure based on the mass accuracy of the peaks. Interestingly, hepta-acylated peaks at *m/z* values 1950.35 and 1970.36 were not detected in the GFP-positive (GFP^+^) or negative (GFP^−^) cells, suggesting that intracellular bacteria in GFP-positive cells were enriched in hexa-acylated lipid A (*m/z* 1740.15), whereas extracellular bacteria displayed both hexa- and hepta-acylated lipid A structures.

### *V. para* VP0213 Functionsas a Late Acyltransferase (LpxM).

We then searched for a putative secondary acyltransferase that could add the acyl chain (either a C14 or C16:1) to the acyl chain at the 2-position of lipid A. Our extensive bioinformatic approaches failed to identify a PagP-like acyltransferase enzyme that has previously been shown to perform this 2-acyl modification (36). However, some bacteria, such as *Acinetobacter baumannii*, have developed a PagP-independent mechanism for synthesizing hepta-acylated lipid A. Specifically, *A. baumannii* encodes a dual acyltransferase LpxM that can transfer C14 acyl chains to both the 3′-acyl and 2-acyl positions to make a hepta-acylated lipid A (37). We speculated that *V. para* might also encode an LpxM homolog to perform such a dual activity. To search for the *V. para* gene responsible for adding the secondary acyl chain at the 3′-position, we searched KEGG (Kyoto Encyclopedia of Genes and Genomes) and identified VP0213 (LpxM) as a potential secondary acyl transferase. Phylogenetic analysis of the protein sequences of LpxM and its homologs in other bacteria is depicted in *SI Appendix*, Fig. S3 and *S1 Methods*.

**Fig. 4.**
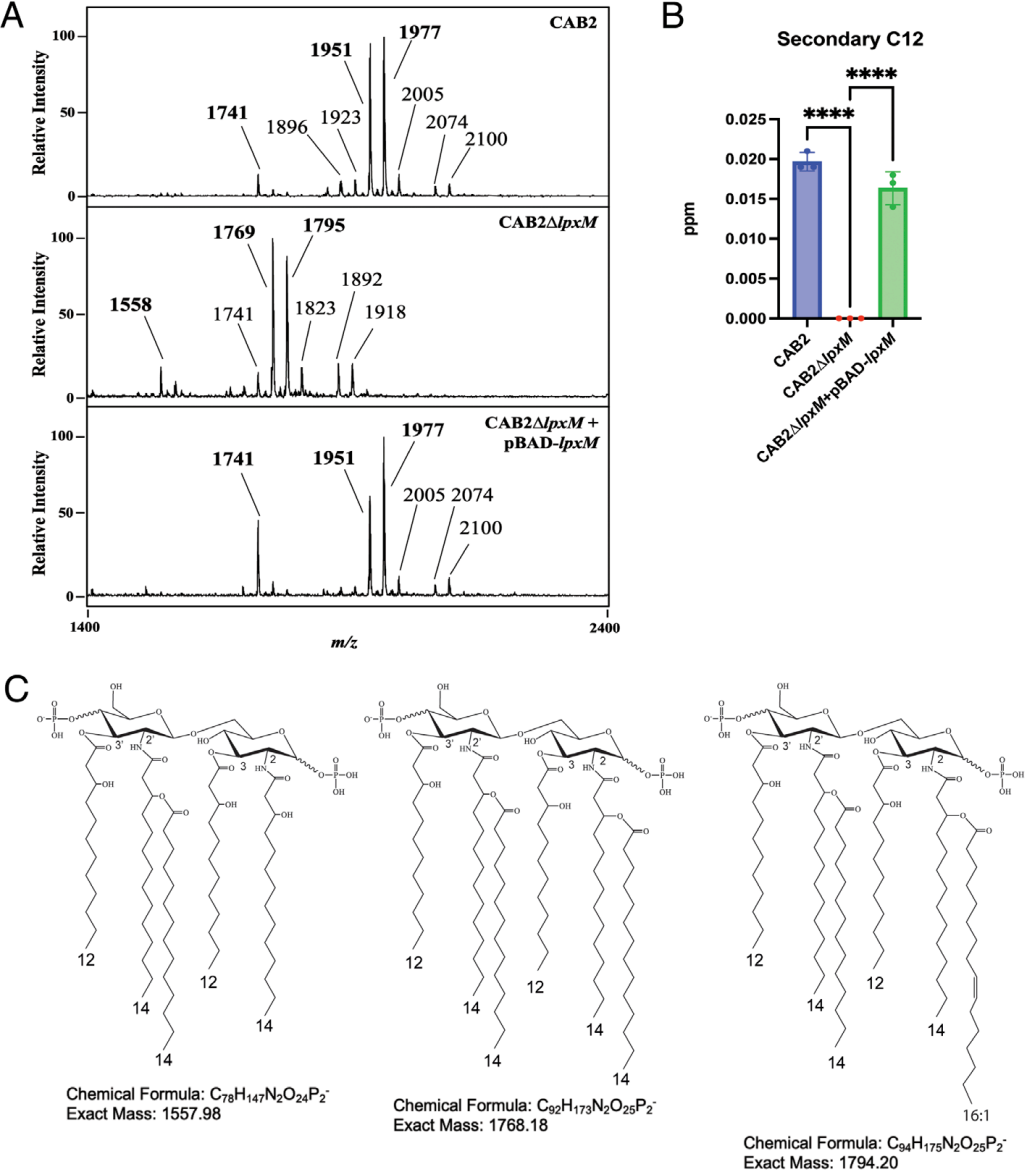
VP0213 (LpxM) of *V. para* functions as a late acyltransferase. (*A*) FLAT-MS spectra of the CAB2, CAB2Δ*lpxM,* and CAB2Δ*lpxM*+pBAD-*lpxM* strains were collected in negative-ion mode using a Bruker microflex LRF. Lipid A peaks (indicated in bold) were present at *m/z* 1,741, 1,951, and 1,977 in the CAB2 and CAB2Δ*lpxM*+pBAD-*lpxM* strains while the CAB2Δ*lpxM* strain shows lipid A peaks at *m/z* 1,558, 1,769, and 1,795. The minor peaks in CAB2 and CAB2Δ*lpxM*+pBAD-*lpxM* strains at *m/z* 2,074 and 2,100 correspond to phosphoethanolamine additions to lipid A at *m/z* 1,951 and 1,977 respectively. Similarly, the minor peaks in CAB2Δ*lpxM* at *m/z* 1,892 and 1,918 are phosphoethanolamine modified lipid A at *m/z* 1,769 and 1,795 respectively. Other minor peaks at *m/z* 1,923 and 2,005 in the CAB2 and CAB2Δ*lpxM*+pBAD-*lpxM* strains and 1,741 and 1,823 in the CAB2Δ*lpxM* were also observed. (*B*) Absence of C12 acyl chain in the CAB2Δ*lpxM* strain and restoration of the C12 acyl chain in the CAB2Δ*lpxM*+pBAD-*lpxM* strain verified by gas chromatography. Statistical significance was calculated using one-way ANOVA. (*C*) Chemical structure of the penta-acylated (*m/z* 1,769) and hexa-acylated (*m/z* 1,795) lipid A in the CAB2Δ*lpxM* strain are as deduced by timsTOF.

To functionally investigate the role of LpxM, we engineered an in-frame deletion of *lpxM* from the CAB2 strain of *V. para* to generate a CAB2Δ*lpxM* mutant (Table 1). As shown in Fig. 4*A*, FLAT mass spectra show that deletion of *lpxM* resulted in lipid A peaks at *m/z* 1,558, 1,769, and 1,795 in the CAB2Δ*lpxM* strain as compared to 1,741, 1,951, and 1,977 in the wild-type CAB2 strain. The minor peaks in CAB2 and CAB2Δ*lpxM*+pBAD-*lpxM* strains at *m/z* 2,074 and 2,100 are 123 *m/z* above the lipid A at *m/z* 1,951 and 1,977, respectively, indicating the addition of phosphoethanolamine to the 1 or 4’ terminal phosphates of lipid A (38). Similarly, the minor peaks in CAB2Δ*lpxM* at *m/z* 1,892 and 1,918 are phosphoethanolamine modified lipid A at *m/z* 1,769 and 1,795, respectively. GC analysis of the distribution of the acyl chains showed absence of C12 acyl chain in the CAB2Δ*lpxM* strain (Fig. 4*B*). The peaks at 1,558, 1,769, and 1,795 in the CAB2Δ*lpxM* strain correspond to penta-acylated (1,558) and hexa-acylated (1,769, 1,795) lipid A with the absence of the C12:0 secondary acyl chain linked to the 3′-position of lipid A (Fig. 4*C*). These structures were confirmed by FLAT^n^ (*SI Appendix*, Fig. S4). We found that deletion of *lpxM* in the CAB2Δ*lpxM* strain did not affect the C14 or C16:1 acyl chains linked to the acyl chain at the C2-position. This observation was further validated by GC-FID which showed no significant differences for the C2 linked secondary acyl chains (*SI Appendix*, Fig. S5). This result suggests that VP0213 (LpxM) functions as a secondary acyltransferase capable of adding a C12 acyl chain linked to the secondary acyl chain at the 3′-position of the lipid A and does not display dual-acyl transferase activity like the *A. baumannii* LpxM. To further validate the role of LpxM as a secondary acyltransferase, we exogenously expressed *lpxM* under the control of an arabinose-inducible promoter in the CAB2Δ*lpxM* strain. Overexpression of LpxM restored the secondary C12:0 acyl chain linked to the acyl chain at the 3′-position of the lipid A (Fig. 4 *B* and *C*).

Due to the unique structures displayed by the CAB2Δ*lpxM* mutant, we wanted to determine whether the altered lipid A structure resulted in phenotypic changes during infection. First, to assess whether deletion of *lpxM* can affect bacterial growth or morphology, we performed a growth curve and CFU determination of these strains. As observed in *SI Appendix*, Fig. S6 *A* and *B*, we did not observe any significant difference in growth of these strains. We also performed confocal microscopy with the strains constitutively expressing GFP on a plasmid and saw no observable differences in the morphology of the CAB2 strain, the mutant CAB2Δ*lpxM* or the complemented CAB2Δ*lpxM* +pBAD-*lpxM* strain during standard growth conditions. However, upon inducing stress, such as treatment with polymyxin antibiotic, which targets the lipid A component of LPS (39), CAB2Δ*lpxM* bacteria exhibited a filamentous phenotype, which disappeared when LpxM was complimented in trans in the CAB2Δ*lpxM* +pBAD-*lpxM* strain (*SI Appendix*, Fig. S6*C*). These results indicate that underacylated lipid A resulting from a lack of LpxM correlates with a weakened outer membrane, making the CAB2Δ*lpxM* strain vulnerable to antimicrobial insults such as polymyxin.

### The Secondary Acyl Chain Synthesized by LpxM contributes to TLR4 activation.

**Fig. 5.**
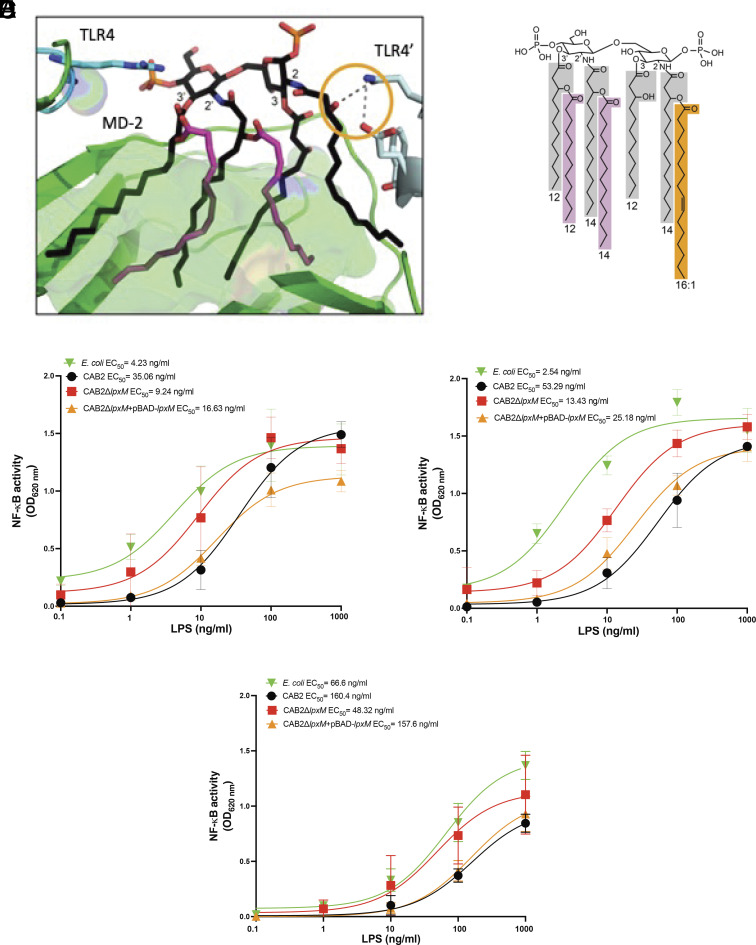
Purified LPS of *V. para* strains differentially stimulate TLR4. (*A*) Lipid A binding pocket in MD-2. MD-2 (green) forms a heterodimer with TLR4 (cyan) and TLR4′ (light cyan) in PDB 3fxi. A large hydrophobic binding pocket in MD-2 (green surface) accommodates 5 of 6 acyl chains from *E. coli* lipid A (black sticks, with secondary acyl chains colored magenta). The R2 primary chain binds at the interface of MD-2 and TLR4′, with the 3-hydroxyl forming a hydrogen bond with a residue from TLR4′ (orange circle). (*B*) *V. para* lipid A includes an additional secondary acyl chain bound to the R2 3-hydroxyl. NF-κB reporter cells expressing (*C*) human-TLR4 or (*D*) mouse TLR4, CD14, and MD2 or (*E*) endogenous TLR4 were stimulated with five-log concentration of LPS from the control strain of *E. coli* (green), wild-type *V. para* CAB2 (black), CAB2Δ*lpxM* (red), and CAB2Δ*lpxM*+pBAD-*lpxM* (orange). After 18 h, NF-κB driven secretion of SEAP was measured colorimetrically at OD 620 nm. The graph was plotted as the mean ± SD of biological duplicates and analyzed using GraphPad Prism version 10 (La Jolla, CA). The EC_50_ values were calculated using the log (agonist)-versus-response (three parameters) for each LPS tested.

To understand the biological implication of altered lipid A structure in the CAB2Δ*lpxM* strain, we first determined the binding of LPS to the extracellular TLR4 complex. In a human TLR4 MD-2 complex structure bound to the *E. coli* LPS agonist, five out of six acyl chains from lipid A bind deep within a hydrophobic pocket in MD-2. The sixth R2 acyl chain binds at the interface of MD-2 and TLR4, forming a hydrogen bond with a residue from TLR4 (Fig. 5*A*). This interaction provides key contacts for productive TLR4 receptor dimerization (Fig. 5*A*, orange circle) and supports the notion that the optimal number of acyl chains from LPS needed for receptor activation is six (40). As observed in previous experiments (Fig. 1*B*), the majority of *V. para* lipid A structure includes an additional secondary acyl chain bound to the 3-hydroxyl of the R2 acyl chain (Fig. 5*B*). The presence of this additional chain would overlap with the TLR4 dimerization interface and prevent the hydrogen bond formation. In this study, we used human HEK-blue hTLR4 and mouse HEK-blue mTLR4 cell lines to determine whether the LPS structure of *V. para* differentially activates TLR4-dependent responses (Fig. 5 *C* and *D*). These cell lines were stimulated with a 5-log concentration of purified LPS for 18 h and NF-κB activation levels were determined by measuring SEAP (secreted embryonic alkaline phosphatase) levels colorimetrically. LPS from *E. coli* was used as a positive control. To establish the degree of activation of TLR4, we determined the EC_50_ (half maximal effective concentration) which is inversely proportional to TLR4 activation. We find that LPS from the CAB2 strain with a higher EC_50_ (EC_50_ = 35.06 ng/mL) weakly activated human TLR4 signaling as compared to LPS from *E. coli* with a lower EC_50_ (EC_50_ = 4.23 ng/mL) in the HEK-blue hTLR4 cell lines (Fig. 5*C*). The LPS from the CAB2Δ*lpxM* strain exhibited a higher level of TLR4 activation due to a lower EC_50_ (EC_50_ = 9.24 ng/mL) as compared to the wild-type CAB2 strain with a comparatively higher EC_50_ (EC_50_ = 35.06 ng/mL). Complementation of LpxM in the CAB2Δ*lpxM +*pBAD-*lpxM* strain led to restoration of SEAP activity similar to wild-type levels (EC_50_ = 16.63 ng/mL) (Fig. 5*C*). Comparable results were observed with mouse TLR4 in the HEK-blue mTLR4 cell lines (Fig. 5*D*) with the EC_50_ of CAB2 (53.29 ng/mL) indicating lower level of TLR4 activation as compared to EC_50_ of *E. coli* (2.54 ng/mL). The EC_50_ values of CAB2Δ*lpxM* (13.43 ng/mL) showed a higher degree of TLR4 activation than the wild-type strain and this activation was partially reduced in the CAB2Δ*lpxM +*pBAD-*lpxM* strain (EC_50_ = 25.18 ng/mL). We also validated these results in the human THP1-dual cell lines, which harbor endogenous levels of TLR4 (Fig. 5*E*). The EC_50_ of *E. coli* was observed to be 66.66 ng/mL, while the CAB2 strain showed a much lower level of activation with EC_50_ 160.4 ng/mL. The EC_50_ values of CAB2Δ*lpxM* and CAB2Δ*lpxM +*pBAD-*lpxM* strain were 48.32 and 157.6 ng/mL, respectively. Together, these findings strongly suggest that the LPS from CAB2 is a weak stimulator of TLR4 signaling in humans and mice, while the LPS generated by CAB2Δ*lpxM* leads to higher levels of activation of TLR4. To further validate that LPS from CAB2Δ*lpxM* induces higher level of TLR4 activation, we performed competitive binding using LPS from CAB2 and CAB2Δ*lpxM* to stimulate HEK-blue hTLR4 cells. We observed that increasing the ratio of CAB2Δ*lpxM* LPS over CAB2 resulted in a lower EC_50_ indicating a higher degree of TLR4 activation (*SI Appendix*, Fig. S7).

### Deletion of *lpxM* Affects Invasion and Replication of *V. para* in Epithelial Cells.

**Fig. 6.**
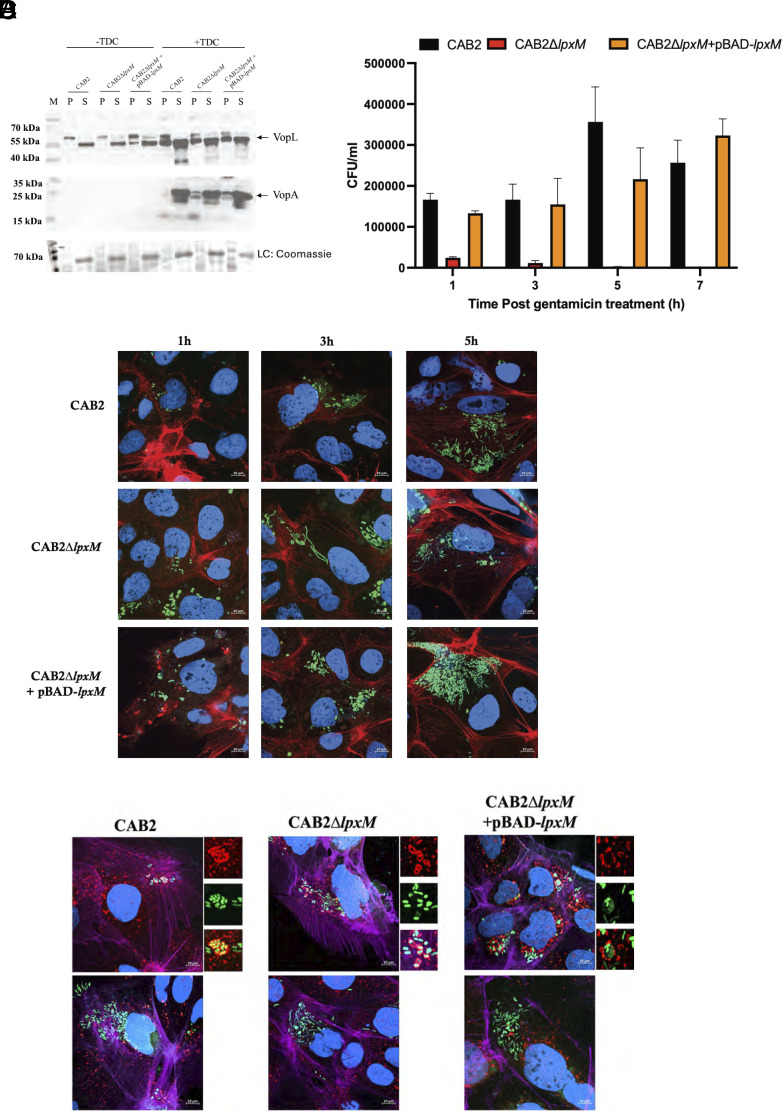
Absence of *lpxM* affects the intracellular replication of *V. para* in Caco-2 but not secretion of effectors. (*A*) Western blot of VopL and VopA in the CAB2, CAB2Δ*lpxM,* and CAB2Δ*lpxM*+pBAD-*lpxM* strains grown in the uninduced or TDC uninduced conditions followed by staining with anti-VopA or anti-VopL antibodies. The T3SS2 effectors VopL and VopA are observed in the supernatant fraction under TDC induced conditions indicating secretion of these effectors. P and S indicate pellet and supernatant fraction, respectively. LC indicates Coomassie loading control. (*B*) Bar graph depicting the gentamicin protection assay of Caco-2 cells infected with CAB2 (black), CAB2Δ*lpxM* (red), and CAB2Δ*lpxM*+pBAD-*lpxM* (orange) for 1.5 h followed by gentamicin treatment to remove extracellular bacteria. At the indicated time points, cells were harvested, and bacterial counts were enumerated by plating on MMM plates. Error bars indicate SD between three independent experiments. (*C*) Confocal micrographs of Caco-2 cells infected with the CAB2, CAB2Δ*lpxM,* and CAB2Δ*lpxM*+pBAD-*lpxM* strains constitutively expressing GFP (green) followed by gentamicin treatment to remove extracellular bacteria. At the indicated time points, cells were harvested and stained with Hoechst to visualize DNA (blue), and Rhodamine phalloidin which stains actin (red). (*D*) Confocal micrographs of Caco-2 cells infected with the CAB2, CAB2Δ*lpxM,* and CAB2Δ*lpxM*+pBAD-*lpxM* strains constitutively expressing GFP (green) followed by gentamicin treatment to remove extracellular bacteria. At the indicated time points, cells were harvested, and immunofluorescence staining was performed using Rabbit anti-EEA1 primary antibody (1:500) and Alexa-fluor goat anti-rabbit secondary antibody (1:500) (red). DNA was stained with Hoechst (blue), and actin was stained using Alexa Flour 680 phalloidin (purple). The subset images indicate colocalization with EEA1.

Since alterations in the outer membrane of bacteria can affect the formation and function of secretion systems, we first tested whether deletion of *lpxM* affects the outer membrane integrity, which would alter the secretion of effector proteins. The *V. para* T3SS2 is induced with the bile salt TDC (8). Once the T3SS2 apparatus is formed, it can then secrete various effectors including VopA and VopL (3). The CAB2, CAB2Δ*lpxM* and CAB2Δ*lpxM+*pBAD-*lpxM* strain*s* were grown in the absence or presence of TDC, and the secretion of effectors VopA and VopL in the supernatant was detected using anti-VopA and anti-VopL antibodies. In the presence of TDC, all the strains expressed and secreted VopL and VopA, indicating that altered lipid A synthesized by the CAB2Δ*lpxM* strain did not interfere with the formation of a functional T3SS2 apparatus (Fig. 6*A*).

The T3SS2 apparatus also contributes to the invasion and replication of *V. para* in nonphagocytic epithelial cells (4). We performed a gentamicin protection assay using CAB2, CAB2Δ*lpxM* and CAB2Δ*lpxM +*pBAD-*lpxM*, to measure the ability of these strains to invade Caco-2 cells. While the CAB2 strain invaded and replicated inside Caco-2 cells, we observed that CAB2Δ*lpxM* exhibited a significant defect in survival at 1 h post gentamicin treatment (PGT), and the mutant bacteria were undetectable by 3 h PGT, indicating that the Caco-2 cells cleared the bacteria following invasion. Complementation of *lpxM* using arabinose induction restored the ability of the CAB2Δ*lpxM* strain to invade and replicate inside Caco-2 cells, albeit not to the levels of the CAB2 bacteria (Fig. 6*B*). We reasoned that Caspase-4 could contribute to restricting the growth of CAB2Δ*lpxM* bacteria inside host cells since Caspase-4 is known to recognize the lipid A of intracellular bacteria and initiate pyroptosis (41). To address this hypothesis, we used Casp4^−/−^ Caco-2 cell line and performed a gentamicin protection assay using the CAB2, CAB2Δ*lpxM,* and CAB2Δ *lpxM+*pBAD-*lpxM* strains. The assay revealed that CAB2Δ*lpxM* was unable to survive in this background, indicating that Caspase-4 is not likely involved in restricting the mutant bacteria in the cytosol of epithelial cells (*SI Appendix*, Fig. S8*A*). We next tested whether the phenotype we observed with Caco-2 cells is observed with infection of other epithelial cells. Using HeLa cells, we observed similar results where the CAB2Δ*lpxM* exhibited a significant defect as compared to CAB2 and CAB2Δ*lpxM*+pBAD-*lpxM* strains (*SI Appendix*, Fig. S8*B*).

Next, we visualized the morphology of the bacteria inside invaded cells. At 1-h PGT, there were no observable morphological differences between the bacteria in invaded cells. However, by 3 h PGT the CAB2Δ*lpxM* bacteria started forming filaments with two- to threefold longer dimension, as compared to the CAB2 or CAB2Δ*lpxM+*pBAD-*lpxM* strains. At 5 h PGT, both CAB2 and CAB2Δ*lpxM+*pBAD-*lpxM* bacteria replicated as expected in the Caco-2 cell cytosol, while CAB2Δ*lpxM* bacteria ceased cell division (Fig. 6*C*). These results indicate that CAB2Δ*lpxM* are attenuated in their ability to divide and are unable to maintain their replicative niche in the cytosol of Caco-2 cells.

We hypothesized that the low intracellular numbers of CAB2Δ*lpxM* in Caco-2 cells might be attributed to its inability to escape from the vacuole. To address this hypothesis, we used immunofluorescence microscopy to visualize intracellular bacteria in Caco-2 cells stained for the early endosomal marker—EEA1 (*SI Appendix*, *S1 Methods*). We observed that the CAB2-GFP, CAB2Δ*lpxM*-GFP and CAB2Δ*lpxM+*pBAD-*lpxM* bacteria were initially enclosed in EEA1 endosomes during early invasion (1 h), and all strains subsequently escaped into the cytosol by 4 h, as evidenced by the absence of EEA1 localization (Fig. 6*D*) (4). Therefore, all strains maintain the ability to escape from endosome and differences are only observed once bacteria enter the cytosol.

## Discussion

The foodborne pathogen *V. para* encodes various virulence factors that can cause enterotoxicity, gastroenteritis, sepsis, and even death (3). The genome of *V. para* encodes adhesins, hemolysins, and secretion systems (T3SS, T6SS) that have been implicated in its virulence. Our lab has discovered several effectors of *V. para* that mediate invasion (VopC) (9), target host signaling (VopA, VopZ) (10, 42), actin cytoskeleton (VopC, VopL, VopT) (43, 44), and egress of the pathogen from the host cell (VPA0226) (12). While much is known about the role of hemolysins and secretion system effectors in the pathogenesis of *V. para*, many unknowns remain, including mechanisms used by *V. para* to escape the host innate immune responses. While many, including ourselves, have focused on how effectors cripple or manipulate the host immune system, how a bacterial pathogen might evade this system altogether is less studied. One of the virulence factors, namely LPS embedded in the outer membrane of Gram-negative bacteria, plays a key role during host–pathogen interaction and establishment of infection. Herein, we focused on *V. para* LPS and observed that *V. para* produces an LPS that masks itself from the innate immune system, allowing invasion into host cells.

In this study, we have successfully employed the FLAT and FLAT^n^ techniques to determine the lipid A structure of *V. para*. This method has been used previously for defining lipid A structures in diverse bacteria (38, 45, 46). Herein, we used this technique to determine the structure of lipid A from *V. para* under regular growth conditions and found that instead of synthesizing a hexa-acylated lipid A, *V. para* predominantly synthesized a hepta-acylated lipid A species, in addition to a minor hexa-acylated form. The hexa-acylated lipid A of *V. para* (*m/z* 1740.15) identified in our study differs from the hexa-acylated lipid A (*m/z* 1765.2) reported by other groups (27, 31). The *V. para* lipid A structure is closely related to that from *V. cholerae,* except for the presence of a hydroxyl group in the secondary acyl chain linked to the 3′ position in *V. cholerae* (47). In addition, we have identified two hepta-acylated structures in *V. para* (*m/z* 1950.35 and 1976.36) that have not been reported before.

*V. para* is unique for containing two distinct hepta-acylated lipid A structures, which consists of either a C14 (*m/z* 1950.35) or a C16:1 (*m/z* 1976.36) acyl chain linked to the acyl chain at the C-2 position (Fig. 1*D*). While the C14 side chain at the C-2 position has been observed in other bacteria (37), the presence of an unsaturated C16:1 chain at this specific position is unique. Hepta-acylated lipid A of *E. coli,* and *S. typhimurium* consist of a C16 fatty-acyl chain linked to the primary acyl chain at the C-2 position, which is facilitated by the palmitoyl transferase PagP (36). Homologs of PagP are also present in *Yersinia* (48)*, Pseudomonas* (49)*, Legionella* (50), and *Bordetella* species (51). However, some bacteria, such as *A. baumannii*, have developed a PagP-independent mechanism for synthesizing hepta-acylated lipid A. Specifically, *A. baumannii* encodes a dual acyltransferase LpxM that can transfer C12 acyl chains to the 2 and 3′ positions to make a hepta-acylated lipid A (37). Since we could not identify a homolog of PagP in *V. para*, we speculated if *V. para* LpxM could also possess a dual acyltransferase activity. However, the deletion of *lpxM* removed only the secondary acyl chain linked at the 3′-position, and did not affect the addition of the C14/C16:1 acyl chains at the C-2 position, indicating that *V. para* likely possesses an alternate unknown enzyme that harbors the unique acyltransferase activity.

Characterization of lipid A from host-invaded intracellular bacteria has revealed significant heterogeneity among bacterial species, particularly during in vivo growth conditions. Recent studies utilizing advanced mass spectrometry techniques have demonstrated that lipid A can undergo structural modifications depending on the host environment, affecting bacterial virulence and immune interactions. Specifically, *S. flexneri* isolated from HeLa cells, hypoacylate their lipid A and synthesize tri-, tetra-, and penta-acylated lipid A. This hypoacylation of intracellular LPS reduces its potential to stimulate an immune response (52). On the other hand, intracellular *Rickettsia rhipicephali* and *Rickettsia parkeri* purified from Vero76 cells synthesize hexa-acylated lipid A (46). The *Rickettsia* are evolving to incorporate shorter 2′ secondary acyl chains into their lipid A, although the driving force for this structural alteration remains to be determined. In our study, we deduced the lipid A structure of *V. para* from invaded Caco-2 cells and revealed that intracellular *V. para* synthesizes only hexa-acylated lipid A (*m/z* 1740.15), suggesting the presence of uncharacterized enzymes that may be downregulated under intracellular conditions or that could remove the seventh acyl chain during replication.

Based on bioinformatic analysis we tested whether LpxM plays a role in modifying *V. para* LPS. In the *V. para lpxM* deletion mutant, we observed a penta-acylated (*m/z* 1,558) and hexa-acylated lipid A (*m/z* 1768.18 and 1794.20), with the notable absence of a secondary acyl chain linked at the 3′ position. Deletion of *lpxM* did not affect the growth or morphology of the bacteria in growth media, however, when challenged with an antimicrobial, bacteria filamented, indicative of increased membrane permeability. While filamentation of the *lpxM* mutant upon exposure to polymyxin has not been shown before, resistance to polymyxin has been attributed to the presence of the secondary C14 acyl chain synthesized by LpxM in bacteria such as *E. coli* and *S. typhimurium* (53).

To date, there have been no studies examining the role of *V. para* lipid A structure in activating the innate immune response. Our findings indicate that *V. para* LPS, which contains hexa- and hepta-acylated lipid A, is nearly two orders of magnitude less potent than *E. coli* at activating either the human or mouse TLR4. Based on modeling, the presence of secondary acyl chains at the C2 position in the hepta-acylated *V. para* lipid A structures pose a steric problem for TLR4 dimerization (Fig. 5 *A* and *B*) and preventing NF-κB activation (Fig. 5 *C*–*E*). Therefore, the presence of two hepta-acylated *V. para* lipid A structures, which is one acyl chain larger than the optimal hexa-acylated LPS from other bacteria, may provide a strategy for this pathogen to evade the immune response initiated by extracellular LPS recognition.

The CAB2Δ*lpxM* mutant LPS lacking the 3′ secondary acyl chain in its lipid A exhibits increased NF-κB activation as compared to the wild-type CAB2 strain LPS. The MD-2 hydrophobic pocket optimally accommodates six acyl chains (40). Accordingly, deletion of the acyltransferase *msbB* (*lpxM*) in *E. coli* (54) and the *lpxN* mutant from *V. cholera*, where removal of the R3′ chain leads to a suboptimal total number of five acyl chains, shows resistance to TLR4 activation (47). Alternately in *Y. pestis*, LPS that loses secondary acylation of R2′ (LpxP mutant) is more resistant to TLR4 activation than is loss of that from R3′ (MsbB mutant), suggesting some secondary acyl chain position specificity for hypoacylated LPS in TLR4 activation (55). Potentially the increased R3′ acyl chain length (C16:1) in *Y. pestis* lipid A might fill the pocket better than the R2′ acyl chain (C12) and account for some of the observed specificity. In case of *V. para*, the CAB2Δ*lpxM* mutant lipid A is no longer able to synthesize hepta-acylated lipid A, leading to the production of the optimal number of six acyl chains accommodated by the MD-2 hydrophobic pocket. It is possible that the overall number of acyl chain numbers in *V. para* lipid A is a critical component for its ability to activate the TLR4/MD-2 mediated proinflammatory response. Based on the competitive binding experiments using LPS from the wild-type CAB2 and CAB2Δ*lpxM* to stimulate HEK-blue-hTLR4 cells, where the LPS from the CAB2 strain was kept constant and an increased concentration of LPS from the CAB2Δ*lpxM* strain was added, we saw a shift in the activation curve, indicating increased binding of the lipid A from CAB2Δ*lpxM* strain, resulting in lower EC_50_ values indicating higher levels of TLR4 activation (*SI Appendix*, Fig. S7). This suggests that LPS from CAB2Δ*lpxM* consisting of penta- and hexa-acylated lipid A induces more effective activation of TLR4 as compared to hexa- and hepta-acylated lipid A synthesized by the wild-type CAB2 strain. Finally, the ratio of C12 and C14 side chains differ in their ability to activate TLR4 (1:5, inducing; 3:3, noninducing) (Fig. 2), supporting the notion that the fidelity of these receptors is tempered by the binding affinities of ligands.

The hepta-acylated lipid A structures that are speculated to help *V. para* evade extracellular TLR4 LPS sensing and immune activation were not detected in the intracellular pathogen (Fig. 3 *B* and *C*). This observation suggests that hepta-acylated lipid A containing R2 secondary acylation aids in evasion of the extracellular immune response, and that once the bacteria invade and replicate within host cells, the hepta-acylated lipid A is no longer required. We have observed that *V. para* lacking LpxM contains a dampened ability to invade and replicate inside intestinal epithelial cells. The loss of the R3′ secondary acyl chain provided by LpxM prevents the ability to synthesize lipid A structures containing six acyl chains under intracellular conditions, implying that hexa-acylated lipid A structures are important for *V. para* invasion and replication in intestinal epithelial cells. One known mediator of immune response to intracellular LPS is Caspase 4/5. However, we find that Caspase 4 does not play a role in this process since its absence does not result in increased intracellular survival of CAB2Δ*lpxM* (*SI Appendix*, Fig. S8*A*). In *Y. pestis*, the 3′ secondary acyl chain of lipid A is required for activation of murine Casp11-dependent cell death (55). Potentially the lack of response of the LpxM mutant to Casp4 may result from a loss of interaction like that observed in *Y. pestis*. Nevertheless, an alternate immune response could be responsible for the lack of virulence observed for this mutant, such as an increased susceptibility to oxidants or other small molecules (56). The presence of LpxM is indeed essential for virulence of other bacteria as exemplified by reports in literature: *lpxM* mutants in *Shigella* were found to be defective in invasion and spread in epithelial cells (57), virulence of *A. baumannii* in the *Galleria mellonella* model requires LpxM (58), and *E. coli lpxM* mutants had a reduced invasion and survival in avian macrophages (59). Future studies can address mechanisms that contribute to the decreased survival of these mutant bacteria within host cells. In support of this, Che et al., recently reported that deletion of *msb*B (*lpxM)*, affected the virulence of *V. para* in the *Tetrahymena* and shrimp model (60).

**Fig. 7.**
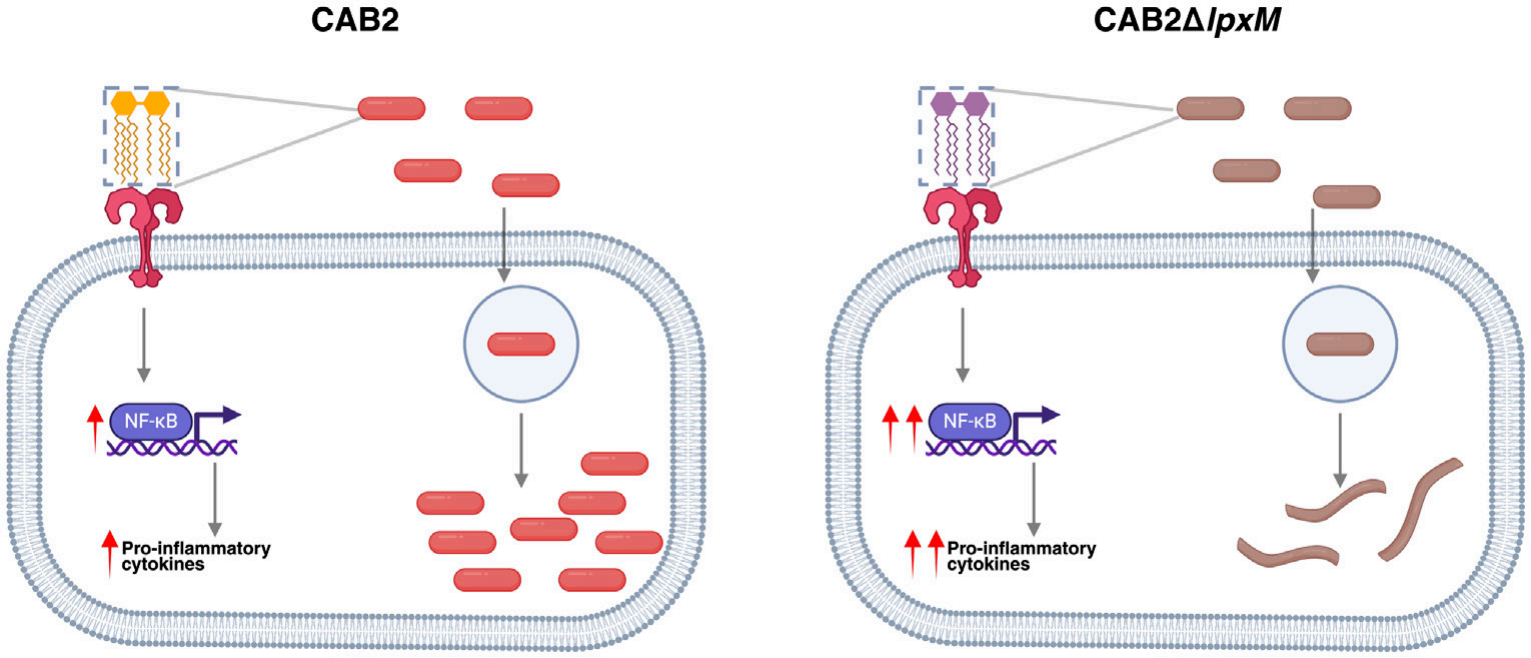
Model depicting the stimulation of immune response and replicative niche of bacteria inside host cell. The predominantly hepta-acylated lipid A in the wild-type CAB2 strain of *V. para* (yellow) weakly binds to the surface exposed TLR4 and leads to a lower level of NF-κB activation. Once these bacteria invade host cells, they maintain a replicate niche in the cytosolic compartment of the host cell. On the other hand, the mutant CAB2Δ*lpxM* bacteria generate a predominantly hexa-acylated lipid A (purple) that is a potent activator of TLR4. Although these bacteria invade host cells similar to the wild-type bacteria, they are recognized by the host, form stress filaments, and are eventually cleared by the host (Created in BioRender. Jaishankar, J. (2025) https://BioRender.com/p05h943).

In this study, we have elucidated the structure of *V. para* lipid A and its role in activating the innate immune response. We have identified a hepta-acylated lipid A structure produced by *V. para* that has not been previously reported in the literature. Deletion of *lpxM* in *V. para* increases its extracellular detection by the TLR4 pathway as well as attenuates its intracellular replication inside epithelial cells (Fig. 7). Consequently, *V. para* lipid A has evolved to maintain a structure that enables evasion of the TLR4 immune responses and allows survival and replication inside host cells. Our work highlights a unique mechanism that *V. para* uses for a successful infection, revealing a unique aspect of *Vibrio* pathogenesis.

## Materials and Methods

### Bacterial Strains, Media, and Growth Conditions.

The *V. para* CAB2, CAB3, and CAB4 strains were derived from POR1 (clinical isolate RIMD2210633 lacking TDH toxins, generously gifted by Drs. Tetsuya Iida and Takeshi Honda) (61). *V. para* non-AHPND strain A2 and AHPND strains strain A3 and D4 were generous gifts from Dr. Donald Lightner at the University of Arizona, and are described elsewhere (34). All the strains were grown in MLB [Lysogenic Broth (LB) medium supplemented with 3% NaCl (w/v) at 30 °C for routine cultivation. When necessary, antibiotics-spectinomycin 50 µg/mL and kanamycin 250 µg/mL were added to maintain the plasmids.] For lipid A structural detection, *E. coli* (O157:H7) was grown in LB and *V. para* strains were grown in minimal marine media containing MgSO_4_ as described previously (62).

### Generation of Mutants and Complementation.

For in-frame deletion of *lpxM*, allelic exchange using suicide vector, pDM4 was employed. Briefly, nucleotide sequences 1 kb upstream and downstream of the respective gene were amplified using primer pairs JJ184 (5′-

GTCACAGCTAGCCCACCGTTTTACATGCAGCAAGAAG

-3′)/JJ185 (5′- GGTAACTAGACACGCCATGAAAAAAATTCTAATTATTGGCCC

-3′) and JJ186 (5′- ATAATTAGAATTTTTTTCATGGCGTGTCTAGTTACCTATCCGTC

-3′)/JJ187 (5′ CTGACACTCGAGCAAGTACATGATGCTAAACAACTATG

-3′), the fragments joined by overlap PCR using primers JJ184/JJ187), digested with NheI and XhoI and cloned into suicide vector pDM4 digested with the same enzymes. After successful cloning, the *E. coli* S17 strain containing the plasmid was conjugated to *V. para* CAB2 strain and the transconjugants were selected on minimal marine medium (MMM) agar containing 25 µg/mL chloramphenicol. For curing *sacB*-containing pDM4, the conjugants were counterselected on MMM agar containing 15% (w/v) sucrose. Deletion was confirmed by PCR and sequencing. For reconstitution of CAB2Δ*lpxM*, the sequence coding for *vp0213* was amplified using primers JJ221 (5′- CAGTCACTGCAGATGACAGCAAAACGTGACGACTACGA

-3′)/JJ222 (5′- CTGACTGAATTCTCATTTTTCAACTTGCCAGTCAGTG

-3′) and cloned into pBAD-Fix between the Pst1 and EcoR1 restriction sites. The resultant construct was transformed to CAB2Δ*lpxM* via triparental conjugation using *E. coli* DH5É’containing pRK2073 (Kan^R^). The transconjugants were selected on MMM agar containing 250 µg/mL kanamycin. Expression of LpxM was induced by adding 0.02% arabinose.

### FLAT.

Lipid A structural analysis was performed using FLAT as previously described (33, 38) (*SI Appendix*, *S1 Methods*).

### MS/MS Experimental Conditions and Gas Chromatography.

Tandem mass spectrometry was utilized alongside FLAT to solve the chemical structure of lipid A, as previously described (33). Lipid A fatty acids were converted to fatty acid methyl esters (FAMEs) and analyzed using gas chromatography paired with flame ionization detection (GC-FID) as previously described (63). The detailed methods are described in *SI Appendix*, *S1 Methods*.

### FACS.

To collect intracellular bacteria for lipid A analysis, CAB2 transformed with pMW-GFP was used at an MOI of 10 to infect Caco-2 cells (cultured in ten 6-well plates) for 1.5 h. After 4 h of addition of gentamicin (100 µg/mL) to kill extracellular bacteria, the infected Caco-2 cells were washed twice with PBS, trypsinized, and centrifuged at 1,000 rpm for 5 min to collect the cells. The cells were then washed with PBS and resuspended in ice-cold PBS. FACS was used to separate host cells containing intracellular bacteria (GFP-positive, ~2% of total input) from bystander host cells without bacteria (GFP-negative, ~45% of total input), using a specific gating strategy to collect individual cell populations. The sorted cells were fixed, harvested, and directly used for lipid A analysis The samples were run on BD-FACS Aria flow cytometer and FACS Diva 6.0 software was used to collect the GFP-positive and GFP-negative cell populations.

### Isolation of LPS and TLR4 Stimulation.

LPS for cell stimulation studies was purified using a hot phenol/water isolation method as previously described (64). Samples underwent a Folch solution wash to remove contaminating phospholipids (65), followed by an ethanol precipitation to remove contaminating lipoproteins. To determine the TLR4 activation in response to different LPS preparations, reporter cell lines with a secreted alkaline phosphatase gene under the control of the NF-κB promoter were used. The HEK-blue cell lines stably express TLR4, MD-2, and CD14 along with an inducible reporter gene for SEAP. Upon stimulation by LPS, TLR4- mediated NF-κB activation can be measured colorimetrically using QUANTI-Blue, a SEAP detection reagent. (Invivogen). HEK-Blue hTLR4 and HEK-Blue mTLR4 cell lines (Invivogen) were cultured in Dulbecco’s modified Eagle’s medium (DMEM) and THP-1 Dual cell line (Invivogen) were cultured in Advanced RPMI medium; both media were supplemented with 10% Fetal bovine serum (FBS), 1% Penicillin and Streptomycin, and 2 mM-L-glutamine. For TLR4 stimulation, the HEK-blue hTLR4/mTLR4 cell lines were seeded at a density of 45,000 cells/well in a 96-well plate. THP-1 Dual cells were cultured with 50 nM Vitamin D3 for activation and differentiation into macrophage-like cells, prior to TLR4 stimulation. Cells were seeded at a density of 100,000 cells/well in a 96-well plate. LPS stimulations were done for 18 to 20 h with a 5-log dose range and SEAP production was detected using the Quanti-Blue reagent (Invivogen) according to the manufacturer’s instructions.

### Secretion of Effectors VopL and VopA.

Overnight cultures of CAB2, CAB2Δ*lpxM,* and CAB2Δ*lpxM*+pBAD-*lpxM* were subcultured at an OD_600_ of 0.1 in LB media supplemented with 20 mM KCl. 100 µM TDC was added to induce T3SS2. Arabinose was added at a concentration of 0.02% and the bacteria were grown at 37 °C for 3 h. To collect the pellet fraction, bacterial cultures of OD_600_ of 0.5 were pelleted and resuspended in 2X Laemmli buffer. For the secretion fraction, the cultures were normalized to the lowest OD among samples and spun at 4,000 rpm, 15 min at 4 °C. The supernatant was filtered through a 0.22 µm filter into chilled conical tubes. Deoxycholate (150 µg/mL), BSA (1%w/v), and Trichloroacetic acid (7% v/v) were added to the supernatant, mixed by inverting, and protein precipitation was allowed to proceed at 4 °C overnight. The following day, the precipitated proteins were collected by spinning the tubes at 14,000 rpm for 15 min at 4 °C, and the pellet was washed twice with ice-cold acetone. After air-drying for 30 min, the pellet was resuspended in 2X Laemmli buffer. Expression and secretion of VopL and VopA were detected by western blot analysis using rabbit anti-VopL (1:3,000) and rabbit anti-VopA (1:1,000) primary antibodies and donkey anti-rabbit HRP conjugated (1:10,000) secondary antibody. The membrane was developed using Pierce ECL Western Blotting substrate (Thermo Scientific) and visualized using ChemiDoc Imaging System (Bio-Rad).

### Cell Culture and Gentamicin Protection Assay.

Caco-2 cells were cultured in MEM/EBSS with 20% FBS, 1% Penicillin and Streptomycin (PenStrep) for routine culture. For gentamicin protection assay, cells were seeded at a density of 2 × 10^5^ cells/mL in each well in 24-well plates for 24 to 48 h. Before infection, cells were washed twice with PBS and replaced with MEM/EBSS with no FBS or PenStrep. Bacteria were added at an MOI of 10, and the plates were spun at 1,000 rpm for 5 min to sync infection. The infection was allowed to proceed for 1.5 h at 37 °C and 5% CO_2_. The plates were then washed twice with PBS, and MEM/EBSS containing 100 µg/mL gentamicin were added to each well. The plates were removed after 1, 3, 5 and 7 h post–gentamicin treatment and CFU was estimated according to standard protocols. Immunofluorescence and confocal microscopy are described in *SI Appendix*, *S1 Methods*.

## Supplementary Material

Appendix 01 (PDF)

## Data Availability

There are no data underlying this work.
