# Supplementary material for: A critical role for Vibrio parahaemolyticus LPS to mediate evasion of host immune response during infection
Source: Proc Natl Acad Sci U S A. 2025 Aug 13;122(33):e2426547122. doi: 10.1073/pnas.2426547122 (PMC12377781; doi:10.1073/pnas.2426547122)
Supplement: Supplementary file 1 — Appendix 01 (PDF) [file pnas.2426547122.sapp.pdf]

Supporting Information for

A critical role for *Vibrio parahaemolyticus* LPS to mediate evasion of host immune response during infection

Jananee Jaishankar[1,2], Hyojik Yang[3], Ian P O'Keefe[3,4], Brandon M Tenaglia[3], Lisa Kinch[1,2], Robert K Ernst[3]*, Kim Orth[1,2,5]*

Email: kim.orth@utsouthwestern.edu, rkernst@umaryland.edu

This PDF file includes:

S1 Methods
Figures S1 to S8
References

S1 Methods

Fast Lipid Analysis Technique (FLAT)

For each FLAT experiment, 1 µL of pelleted bacterial slurry was spotted onto a stainless steel 96-well MALDI target plate, followed by the addition of 1 µl of FLAT extraction buffer (0.2 M anhydrous citric acid, 0.1 M trisodium citrate dihydrate, pH 4.5). The FLAT target plate was incubated at 100°C in a humidified heat block for 30 minutes. Bacterial spots were thoroughly washed with endotoxin-free water and air-dried, followed by the addition of norharmane matrix (10mg/mL in 1:2 MeOH:$CHCl_3$ (v:v) – Sigma Aldrich, St. Louis, MO, USA). Mass spectra were collected in negative-ion mode using a Bruker microflex LRF within the 1000-2400 *m/z* range, 300 shots. Agilent ESI Tune Mix was used as an external calibrant. MALDI-TOF MS data were processed and analyzed with flexAnalysis software (version 3.4). All other chemicals were obtained from Sigma-Aldrich unless otherwise noted.

MS/MS experimental conditions

A Bruker Matrix-Assisted Laser Desorption/Ionization trapped ion-mobility spectrometry time-of-flight Mass Spectrometry MALDI (timsTOF) MS was used for FLAT (direct biomass MS for visualization of lipid A and others primary molecular ions) followed by $FLAT^n$ (direct biomass MS/MS for fragmentation and structural analysis of lipid A in negative ion mode). In the MS/MS mode, the precursor ion was chosen by targeting the m/z value to the hundredth of a mass unit. For collision-induced dissociation (CID), typical isolation width and collision energy were set to 4−6 m/z and 100−110 eV, respectively. Agilent ESI Tune Mix was used to calibrate the m/z scale. MALDI parameters in qTOF were optimized to maximize the intensity by tuning ion optics, laser intensity, and laser focus. All spectra were collected at 104 µm laser diameter with beam scan on using 800 laser shots per spot and 70 and 80% laser power, respectively. Both MS and MS/MS data were collected in negative ion modes using 10 mg/mL norharmane matrix in 1:2 MeOH:$CHCl_3$ (v:v) for ionization of lipid. All MALDI (tims TOF) MS and MS/MS data were visualized using mMass (Ver 5.5.0, www.mmass.org) peak picking was conducted in mMass by following parameters: S/N threshold: 3.0, absolute intensity threshold: 1.0, relative intensity threshold: 1.0%, picking height: 50, apply baseline, and apply smoothing. Identification of all fragment ions was determined based on exact mass as predicted by ChemdrawUltra (Ver23.1, PerkinElmer, Waltham MA) (1).

Gas Chromatography

Lipid A fatty acids were converted to fatty acid methyl esters (FAMEs) and analyzed using gas chromatography paired with flame ionization detection (GC-FID) as previously described (2) The detailed methods are described in S1 Materials and Methods. Briefly, bacterial cells were pelleted at mid-log phase and lyophilized overnight. The freeze-dried pellet was incubated at 70°C for 1 hour in 500 µl of 90% phenol and 500 µl of endotoxin-free water (Gibco, Grand Island, New York, USA). Samples were cooled in an ice bath for 5 minutes and centrifuged at 10,000 xg for 10 minutes. The aqueous layer was collected and 500 µl of water was added to the lower (organic) layer and incubated again, repeated twice for a total of three extractions. All aqueous layers were pooled together, and 2 mL of diethyl ether was added, vortexed and centrifuged at 3,000 xg for 5 minutes. The supernatant was decanted, and another 2 mL of diethyl ether was added, vortexed, and centrifuged. The lower (aqueous) layer was collected and lyophilized overnight. LPS fatty acids were converted to FAMEs using 2 M methanolic HCl at 90ºC for 18 h in the presence of 20

µg pentadecanoic acid (Sigma, St Louis, MO) as an internal standard. The resulting FAMEs were extracted twice with hexane and analyzed using a Shimadzu GC-2010 Plus Gas Chromatograph with an AOC-6000 Plus Auto Sampler. Retention times were correlated to fatty acids using GC-FAME standards (Matreya, Pleasant Gap, PA) and quantified by interpolating from a standardized curve for each fatty acid (3) (LabSolutions v5.111).

Phylogenetic tree construction

The sequence for Vibrio parahaemolyticus RIMD 2210633 LpxM (NP_796592) was used with psi-blast (3 iterations, E-value cutoff $1E^{-6}$) to query a library of prokaryotic reference genomes selected by NCBI based on recognition of community standard, medical importance, sequence and annotation quality, and the availability of experimental support. The genomes include accessions that were assembled prior to the NCBI re-annotation project. Identified sequences included LpxM family sequences, as well as related LpxL and LpxP homologs. We generated a phylogenetic tree for identified sequences using NGPhylogeny.fr (4) one click workflow (selecting the programs: mafft multiple sequence alignment (5), BMGE cleaned sequences (6), and PhyML distances for output tree (7). We then limited the set to include LpxM sequences with the dual *Acinetobacter baumannii* LpxM-like sequence as an outgroup. The LpxM tree was rebuilt with the same methods and illustrated with the interactive Tree Of Life (iTOL) online tool (8).

Immunofluorescence and confocal microscopy

Caco-2 cells were seeded at a density of 2*10^5 cells/ml on sterile poly-L-Lysine coated coverslips (Neuvitro Corporation, USA). Following infection with CAB2, CAB2Δ*lpxM* and CAB2Δ*lpxM*+pBAD-*lpxM* containing pMW-GFP and indicated gentamicin treatment times, cells were washed twice with PBS and fixed with 3.2% (v/v) paraformaldehyde for 10 min at room temperature with gentle shaking. Cells were then washed once with PBS, and permeabilized using 0.1% Triton-X-100 for 10 min. For EEA1 staining was performed as described previously (9). Hoechst (1:1000) (Sigma) and rhodamine-phalloidin (1:1000) (Molecular Probes) were used to stain nuclei and actin, respectively. After washing with PBS, the coverslips mounted on to glass slides with ProLong Gold Antifade Mountant and cured overnight. All imaging was performed on a Zeiss LSM 710 confocal microscope.

A

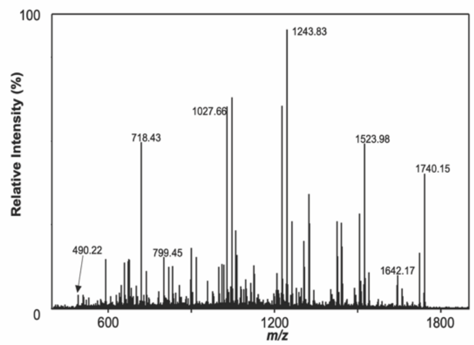

Naming	m/z (theory)	m/z (expt)	Error (Da)
B'_2	1642.17	1642.16	0.01
3α	1523.98	1523.97	0.01
B'_2 + 3'α	1243.83	1243.83	0.00
B'_2 + 3'α + 3α	1027.66	1027.66	0.00
B'_2 + 3'α + 3α + 2'ε	799.45	799.45	0.00
$^{0,4}A_2$ + 3'α	718.43	718.43	0.00
$^{0,4}A_2$ + 3'α + 2'ε	490.22	490.22	0.00

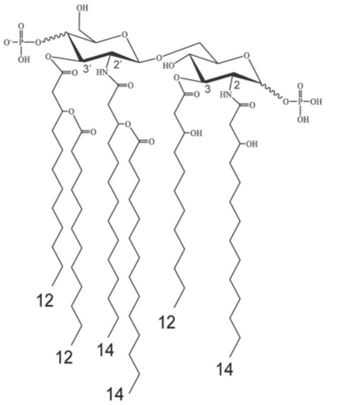

Chemical Formula: $C_{90}H_{169}N_2O_{25}P_2^-$
Exact Mass: 1740.15

B

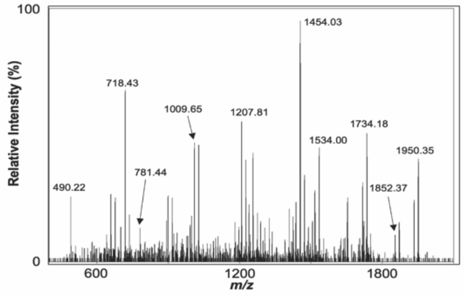

Naming	m/z (theory)	m/z (expt)	Error (Da)
B_2	1852.37	1852.36	0.01
3α	1734.18	1734.18	0.00
3α + 3'ε	1534.00	1534.00	0.00
B_2 + 3'α	1454.03	1454.03	0.00
B_2 + 3α + 3'ε + 2ε	1207.81	1207.81	0.00
B_2 + 3α + 3'α + 2ε	1009.65	1009.65	0.00
B_2 + 3α + 3'α + 2'ε + $2^ε$	781.44	781.44	0.00
$^{0,4}A_2$ + 3'α	718.43	718.43	0.00
$^{0,4}A_2$ + 3'α + 2'ε	490.22	490.22	0.00

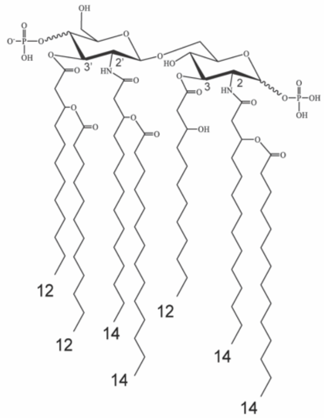

Chemical Formula: $C_{104}H_{195}N_2O_{26}P_2^-$
Exact Mass: 1950.35

C

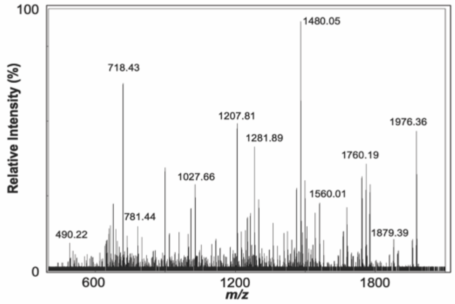

Naming	m/z (theory)	m/z (expt)	Error (Da)
B_2	1879.39	1879.39	0.00
3α	1760.19	1760.19	0.00
3α + 3'ε	1560.01	1560.01	0.00
B_2 + 3'α	1480.05	1480.04	0.01
B_2 + 3β + 3'α	1281.89	1281.88	0.01
B_2 + 3α + 3'ε + 2ε	1207.81	1207.81	0.00
B_2 + 3β + 3'α + $2^ε$	1027.66	1027.66	0.00
B_2 + 3α + 3'α + 2ε + 2'ε	781.44	781.44	0.00
$^{0,4}A_2$ + 3'δ	718.43	718.43	0.00
$^{0,4}A_2$ + 3'δ + 2'ε	490.22	490.21	0.01

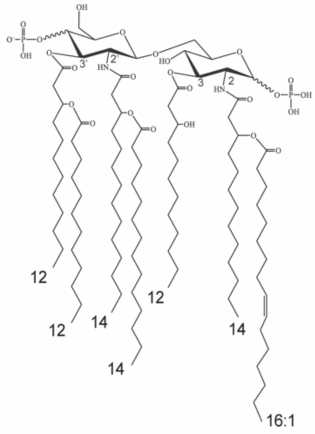

Chemical Formula: $C_{106}H_{197}N_2O_{26}P_2^-$
Exact Mass: 1976.36

Figure S1: FLAT $^{n+1}$ spectra of the lipid A peak at **(A)** 1740.15, **(B)** 1950.35, and **(C)**1976.36 in the wild-type CAB2 strain of *Vibrio parahaemolyticus* and the corresponding lipid A structures.

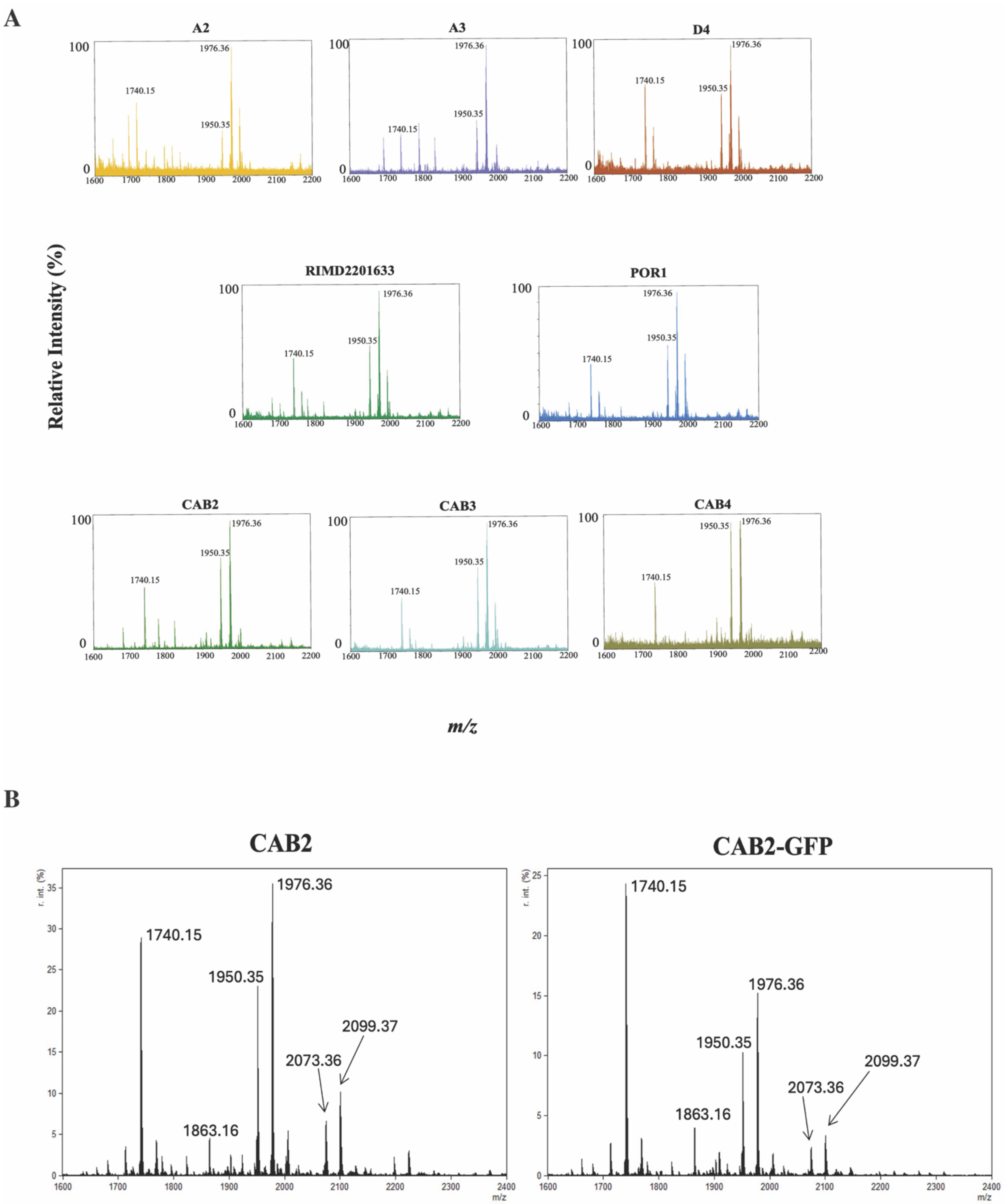

Figure S2: **A**: FLAT^n-MS spectra of *V. parahaemolyticus (V. para)* non-AHPND strain A2 and AHPND strains strain A3 and D4, clinical isolate RIMD2210633, and derivatives POR1, CAB2, CAB3, and CAB4 strains. The peaks at *m/z* 1740.15 correspond to hexa-acylated lipid A, while the peaks at *m/z* 1950.35 and 1976.36 correspond to hepta-acylated lipid A.; **B:** FLAT^n-MS spectra of CAB2 and CAB2-GFP strains indicate no differences in the lipid A peaks.

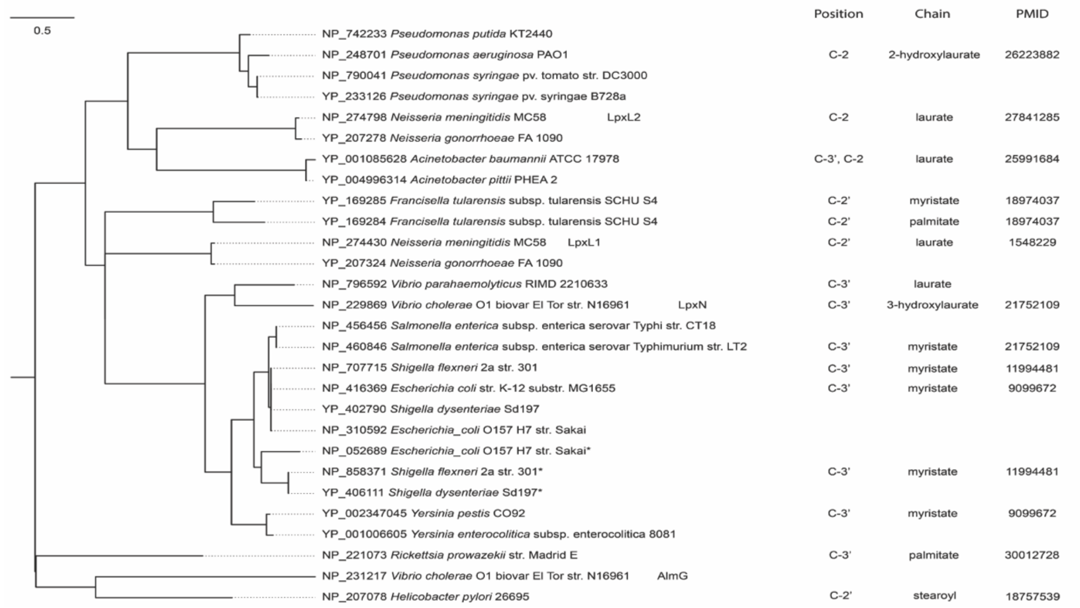

Figure S3: Phylogenetic tree of LpxM homologs in Gram-negative bacteria including *Acinetobacter spp, Vibrio spp, Yersinia spp, Salmonella enterica, E. coli,* and *Shigella flexneri*. The accession number of enzymes capable of adding a secondary acyl chain at the 3' acyl chain hydroxy position of disaccharide are indicated along with the length of primary and secondary acyl chain at the position.

A

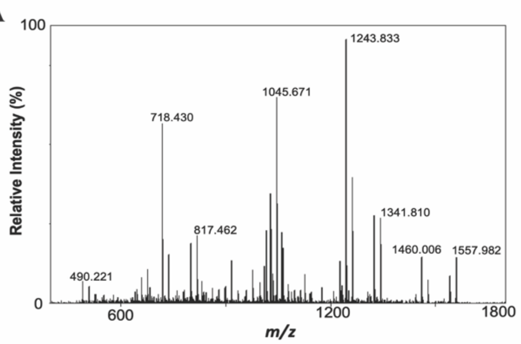

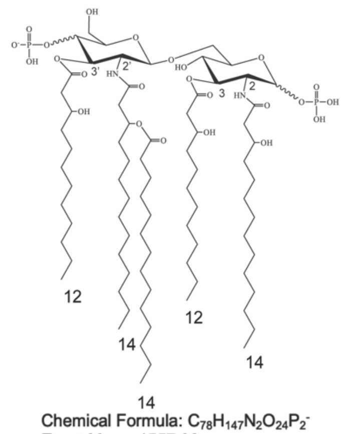

Naming	m/z (theory)	m/z (expt)	Error (Da)
B_2	1460.006	1460.005	0.001
3'α	1341.810	1341.806	0.004
B_2 + 3'α	1243.833	1243.835	0.002
B_2 + 3'α + 3β	1045.671	1045.670	0.001
B_2 + 3'α + 3β +2'ε	817.462	817.462	0.000
$^{0,4}A_2$ + 3'α	718.430	718.430	0.000
$^{0,4}A_2$ + 3'α + 2'ε	490.221	490..223	0.002

B

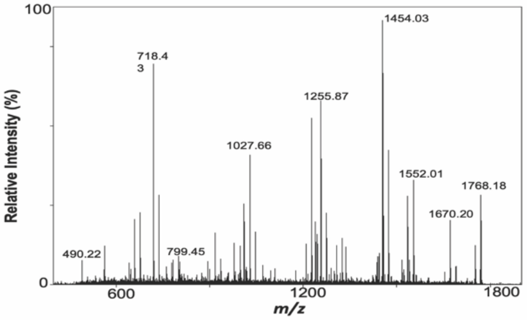

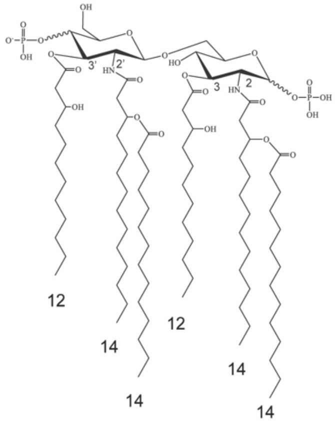

Naming	*m/z* (theory)	*m/z* (expt)	Error (Da)
B_2	1670.20	1670.20	0.00
3α	1552.01	1552.01	0.00
B_2 + 3α	1454.03	1454.03	0.00
B_2 + 3α + 3'β	1255.87	1255.87	0.00
B_2 + 3α + 3'β + 2ε	1027.66	1027.66	0.00
B_2 + 3α + 3'β + 2ε + 2'ε	799.45	799.45	0.00
$^{0,4}A_2$ + 3'α	718.43	718.43	0.00
$^{0,4}A_2$ + 3'α + 2'ε	490.22	490.22	0.00

C

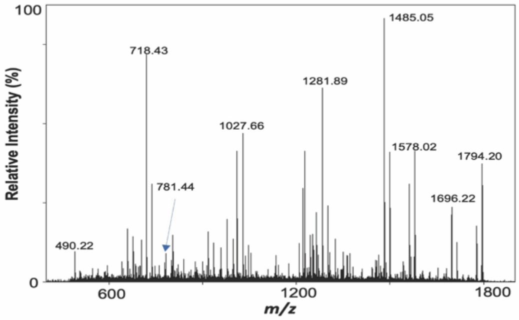

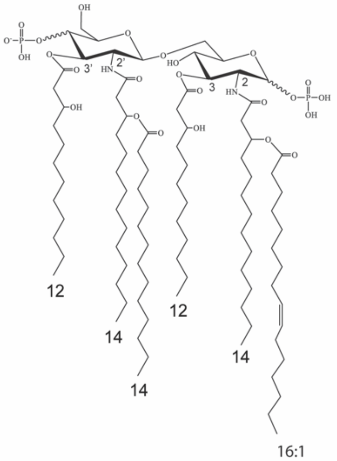

Naming	*m/z* (theory)	*m/z* (expt)	Error (Da)
B_2	1696.22	1696.21	0.01
3α	1578.02	1578.02	0.00
B_2 + 3α	1485.05	1485.04	0.01
B_2 + 3α + 3'β	1281.89	1281.88	0.01
B_2 + 3α + 3'β + 2ε	1027.66	1027.66	0.00
B_2 + 3α + 3'α + 2ε + 2'ε	781.44	781.44	0.00
$^{0,4}A_2$ + 3'α	718.43	718.43	0.00
$^{0,4}A_2$ + 3'α + 2'ε	490.22	490.22	0.00

Figure S4: FLAT $^{n+1}$ spectra of the lipid A peak at **(A)** 1557.98, **(B)** 1768.18, and **(C)** 1794.20 in the mutant CAB2Δ*lpxM* strain of *Vibrio parahaemolyticus* and the corresponding lipid A structures.

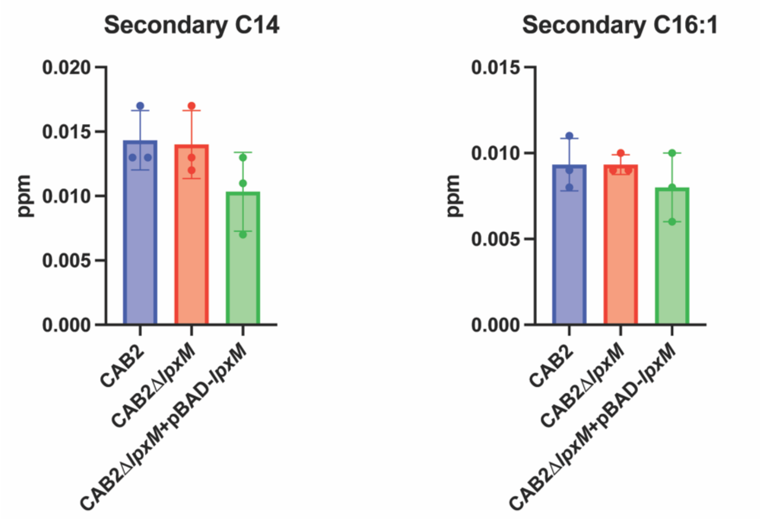

Figure S5: Distribution of C14 and C16:1 acyl chain is not affected in the CAB2Δ*lpxM* strain as compared to CAB2 and CAB2Δ*lpxM*+pBAD-*lpxM*.

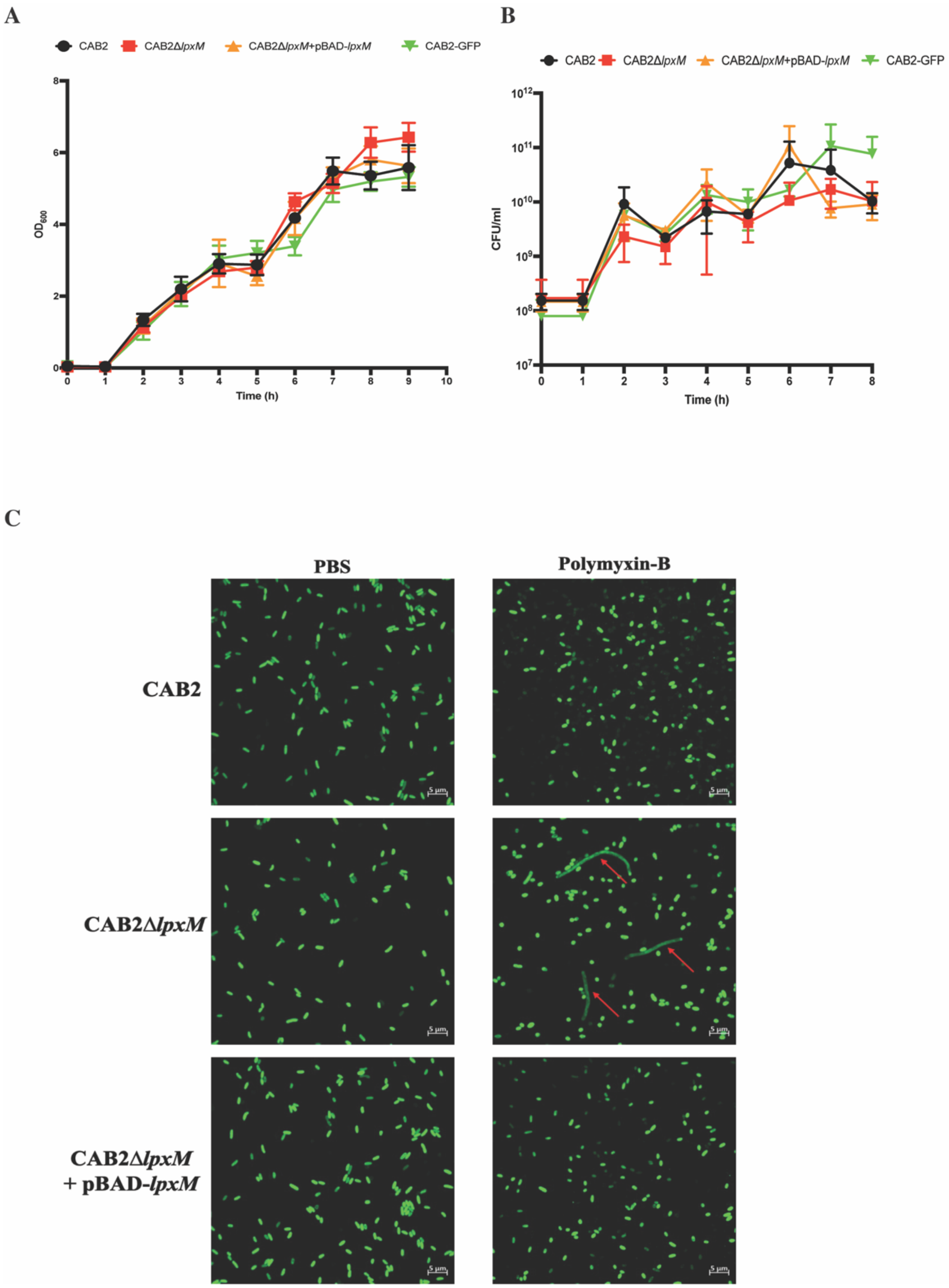

Figure S6: Growth curve (**A**) and CFU (**B**) of CAB2 (black), CAB2-GFP (green), CAB2Δ*lpxM* (red) and CAB2Δ*lpxM*+pBAD-*lpxM* (orange) strains showing no significant difference between the growth rates among the strains **C:** Confocal micrographs of the CAB2, CAB2Δ*lpxM* and CAB2Δ*lpxM*+pBAD-*lpxM* strains harboring a constitutive GFP expressing plasmid grown in MLB media supplemented with (PBS) or of polymyxin-B (50ug/ml). Incubation with polymyxin-B does not affect the morphology of wild-type CAB2 bacteria but leads to filament formation in the CAB2Δ*lpxM* strain. Complementation of *lpxM* on a plasmid and induction with arabinose rescues the phenotype similar to wild-type in the CAB2Δ*lpxM*+pBAD-*lpxM* strain.

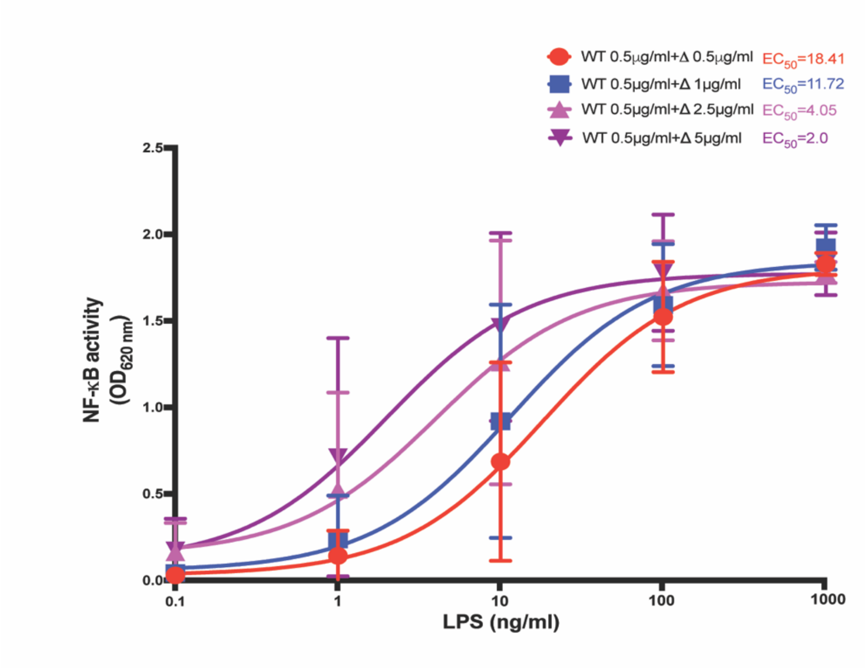

Figure S7: NF-κB reporter cells expressing human-TLR4 was stimulated with five-log concentration of a mixture of LPS from wild-type *V. parahaemolyticus* CAB2 (WT), CAB2Δ*lpxM* (Δ) strains and showed significantly lower EC_{50} values with increasing concentration of LPS from CAB2Δ*lpxM* (Δ) strain indicating higher level of TLR4 activation.

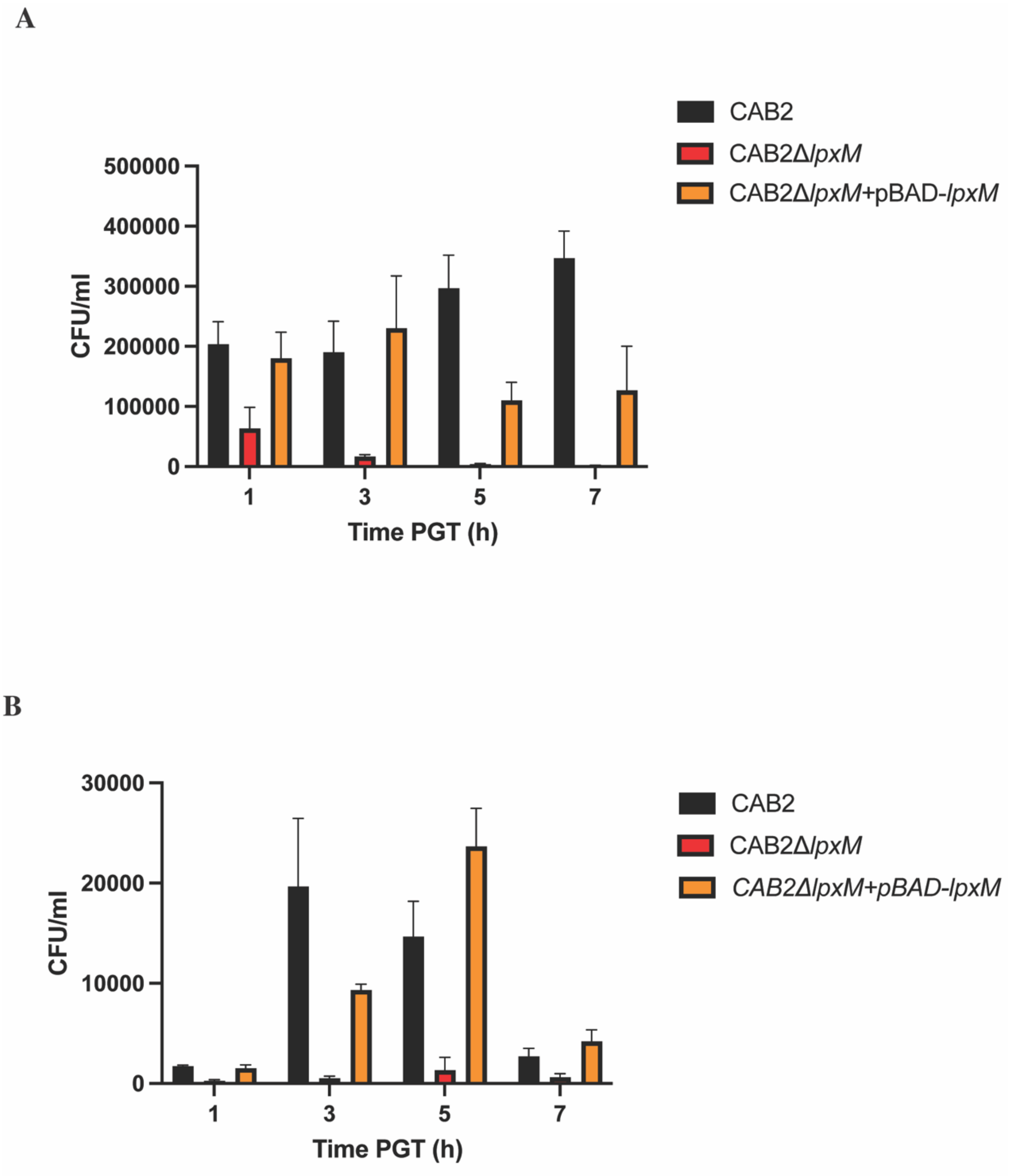

Figure S8: Bar graph depicting the gentamicin protection assay of (**A**) Casp4-/- Caco-2 cells (**B**) HeLa cells infected with CAB2 (black), CAB2Δ*lpxM* (red) and CAB2Δ*lpxM*+pBAD-*lpxM* (orange) bacteria for 1.5 hours followed by gentamicin treatment to remove extracellular bacteria. At the indicated time points, cells were harvested, and bacterial counts were enumerated by plating on MMM plates. Error bars indicate standard deviation between three independent experiments.

References

1. Ghods S, Muszyński A, Yang H, Seelan RS, Mohammadi A, Hilson JS, et al. Frontiers | The multifaceted role of c-di-AMP signaling in the regulation of *Porphyromonas gingivalis*

lipopolysaccharide structure and function. Frontiers in Cellular and Infection Microbiology. 2024/06/12;14.
2. Hittle LE, Powell DA, Jones JW, Tofigh M, Goodlett DR, Moskowitz SM, et al. Site-specific activity of the acyltransferases HtrB1 and HtrB2 in *Pseudomonas aeruginosa* lipid A biosynthesis. Pathogens and Disease. 2015 Jul 29;73(8).
3. Hofstaedter CE, Chandler CE, Met CM, Gillespie JJ, Harro JM, Goodlett DR, et al. Divergent *Pseudomonas aeruginosa* LpxO enzymes perform site-specific lipid A 2-hydroxylation. mBio. 2024-02-14;15(2).
4. Lemoine F, Correia D, Lefort V, Doppelt-Azeroual O, Mareuil F, Cohen-Boulakia S, Gascuel O. NGPhylogeny. fr: new generation phylogenetic services for non-specialists. Nucleic acids research. 2019 Jul 2;47(W1):W260-5.
5. Katoh K, Misawa K, Kuma KI, Miyata T. MAFFT: a novel method for rapid multiple sequence alignment based on fast Fourier transform. Nucleic acids research. 2002 Jul 15;30(14):3059-66.
6. Criscuolo A, Gribaldo S. BMGE (Block Mapping and Gathering with Entropy): a new software for selection of phylogenetic informative regions from multiple sequence alignments. BMC evolutionary biology. 2010 Dec;10:1-21.
7. Guindon S, Dufayard JF, Lefort V, Anisimova M, Hordijk W, Gascuel O. New algorithms and methods to estimate maximum-likelihood phylogenies: assessing the performance of PhyML 3.0. Systematic biology. 2010 Mar 29;59(3):307-21.
8. Letunic I, Bork P. Interactive Tree of Life (iTOL) v6: recent updates to the phylogenetic tree display and annotation tool. Nucleic acids research. 2024 Jul 5;52(W1):W78-82.
9. Santos MdS, Orth K. Intracellular *Vibrio parahaemolyticus* Escapes the Vacuole and Establishes a Replicative Niche in the Cytosol of Epithelial Cells. mBio. 2014-9-9;5(5).
